# Haemodynamics-Driven Developmental Pruning of Brain Vasculature in Zebrafish

**DOI:** 10.1371/journal.pbio.1001374

**Published:** 2012-08-14

**Authors:** Qi Chen, Luan Jiang, Chun Li, Dan Hu, Ji-wen Bu, David Cai, Jiu-lin Du

**Affiliations:** 1 Institute of Neuroscience and State Key Laboratory of Neuroscience, Shanghai Institutes for Biological Sciences, Chinese Academy of Sciences, Shanghai, China; 2 Department of Mathematics and Institute of Natural Sciences, Shanghai Jiaotong University, Shanghai, China; 3 Courant Institute of Mathematical Sciences and Center for Neural Science, New York University, New York, United States of America; Stanford University School of Medicine, United States of America

## Abstract

The brain blood vasculature consists of a highly ramified vessel network that is tailored to meet its physiological functions. How the brain vasculature is formed has long been fascinating biologists. Here we report that the developing vasculature in the zebrafish midbrain undergoes not only angiogenesis but also extensive vessel pruning, which is driven by changes in blood flow. This pruning process shapes the initial exuberant interconnected meshwork into a simplified architecture. Using in vivo long-term serial confocal imaging of the same zebrafish larvae during 1.5–7.5 d post-fertilization, we found that the early formed midbrain vasculature consisted of many vessel loops and higher order segments. Vessel pruning occurred preferentially at loop-forming segments via a process mainly involving lateral migration of endothelial cells (ECs) from pruned to unpruned segments rather than EC apoptosis, leading to gradual reduction in the vasculature complexity with development. Compared to unpruned ones, pruned segments exhibited a low and variable blood flow, which further decreased irreversibly prior to the onset of pruning. Local blockade of blood flow with micro-bead obstruction led to vessel pruning, whereas increasing blood flow by noradrenergic elevation of heartbeat impeded the pruning process. Furthermore, the occurrence of vessel pruning could be largely predicted by haemodynamics-based numerical simulation of vasculature refinement. Thus, changes of blood flow drive vessel pruning via lateral migration of ECs, leading to the simplification of the vasculature and possibly efficient routing of blood flow in the developing brain.

## Abstract 2

This in vivo time-lapse imaging study in zebrafish reveals how changes to brain blood flow drive vessel pruning via endothelial cell migration, and how pruning leads to the simplification of the brain vasculature during development.

## Author Summary

Although the brain comprises only 2% of body weight, it receives 15% of cardiac output and consumes 20% of total body oxygen delivered through its blood vasculature. The brain blood vasculature consists of a highly branched vessel network that is tailored to efficiently deliver oxygen and nutrients to each brain region. However, little is known about how the brain vasculature develops. Using in vivo long-term serial confocal imaging of zebrafish larvae, we analyze this process and find that the developing midbrain vasculature undergoes not only vessel growth but also blood flow-driven vessel pruning. We show that vessel pruning occurs preferentially at loop-shaped vessel segments via the migration of endothelial cells to adjacent unpruned segments; over time, such vessel pruning reduces the complexity of the early primitive midbrain vasculature. We also observe that pruned vessel segments exhibit a lower and more variable blood flow than do unpruned segments and that the local blocking of blood flow triggers vessel pruning. By contrast, increases in blood flow impair vessel pruning. Finally, we show that pruning events can be predicted using a haemodynamics based mathematical model of the midbrain vasculature. These findings demonstrate the existence of brain vessel pruning during development and provide novel insights into the role of haemodynamics in brain vascular refinement.

## Introduction

The brain comprises only 2% of body weight but receives up to about 15% of cardiac output through its blood vasculature [1]. The brain vasculature consists of a highly ramified vessel network with a total vessel length of a few hundred miles in the human and is tailored for efficiently distributing the blood to all brain regions [1],[2]. Abnormalities of the brain vasculature can lead to neurological disorders, including stroke, mental retardation, and neurodegeneration [1],[3],[4]. The brain does not produce vascular endothelial cells (ECs), and the development of its vasculature is initiated by ingression of angiogenic sprouts from surrounding peri-neural vascular plexus [5],[6]. After invading into the brain, angiogenic sprouts become connected to form functional vessel networks via some not well-identified processes, including remodeling [2],[4],[7],[8]. The blood-brain-barrier (BBB), which governs the exchange of material between the brain tissue and vasculature system, is subsequently formed via interactions between ECs, pericytes, astrocytes, and neurons [9],[10].

The process of vessel ingression into the brain has been intensively studied [11]–[17], and the mechanism underlying the BBB formation is also beginning to be elucidated [9],[10],[16],[18],[19]. However, it is poorly understood how the vessel network in the brain develops after vessel ingression, especially the cellular process and underlying mechanism by which the three-dimensional (3-D) network of the brain vasculature is established. This is largely due to the difficulty of imaging large-scale brain vasculature in intact animals. Most studies of brain vessel development were usually performed by using immunohistochemistry on brain slices. While some important insights have been obtained on molecular mechanisms of brain angiogenesis [11]–[17], the formation of 3-D brain vasculature and its dynamic process during development remain to be explored. In addition, because previous studies of hyaloid vasculature have indicated that blood vessels undergo a pruning process during development [20],[21], it is of interest to examine whether vessel pruning occurs in the brain and whether such pruning contributes to the development of the brain vasculature.

To address these questions, we used transgenic zebrafish as an animal model, in which vascular ECs, blood cells, and neurons could be simultaneously labeled by fluorescent proteins. In the same zebrafish larvae, we performed in vivo long-term serial confocal imaging of the midbrain vasculature during 1.5–7.5 d post-fertilization (dpf). This allowed us to trace the developmental process of each vessel segment and monitor changes in the 3-D structure of the entire midbrain vasculature. To quantitatively analyze both morphological and topological properties of the vasculature, we also developed a computer-assisted image processing method. We found that, accompanying its developmental expansion, the midbrain vasculature was remodeled from an initially exuberant interconnected meshwork into a simplified architecture that facilitated efficient blood flow. By tracing the fate of each vessel segment, we demonstrated that this structural simplification was largely due to selective pruning of loop-forming early vessel segments. We then simultaneously monitored changes in both the morphology and blood flow of vessel segments during the pruning process and found that pruned segments exhibited low and variable blood flow, which further decreased irreversibly before the pruning onset. Combining local manipulation of brain blood flow and fluid dynamics-based numerical simulation of realistic 3-D midbrain vasculature, we further revealed that the vessel pruning was triggered by blood flow changes. Furthermore, by tracing single ECs and their nuclei, we found that during vessel pruning, ECs in pruning segments did not undergo apoptosis. Instead, they migrated to and became a part of adjacent unpruned segments, an efficient way for reconstructing the brain vasculature. To our knowledge, these findings demonstrate the existence of vessel pruning during the development of midbrain vasculature and provide insights into the role of haemodynamic forces in shaping the topological structure of the vasculature.

## Results

### Structural Changes of Zebrafish Midbrain Vasculature during Development

**Figure 1:**
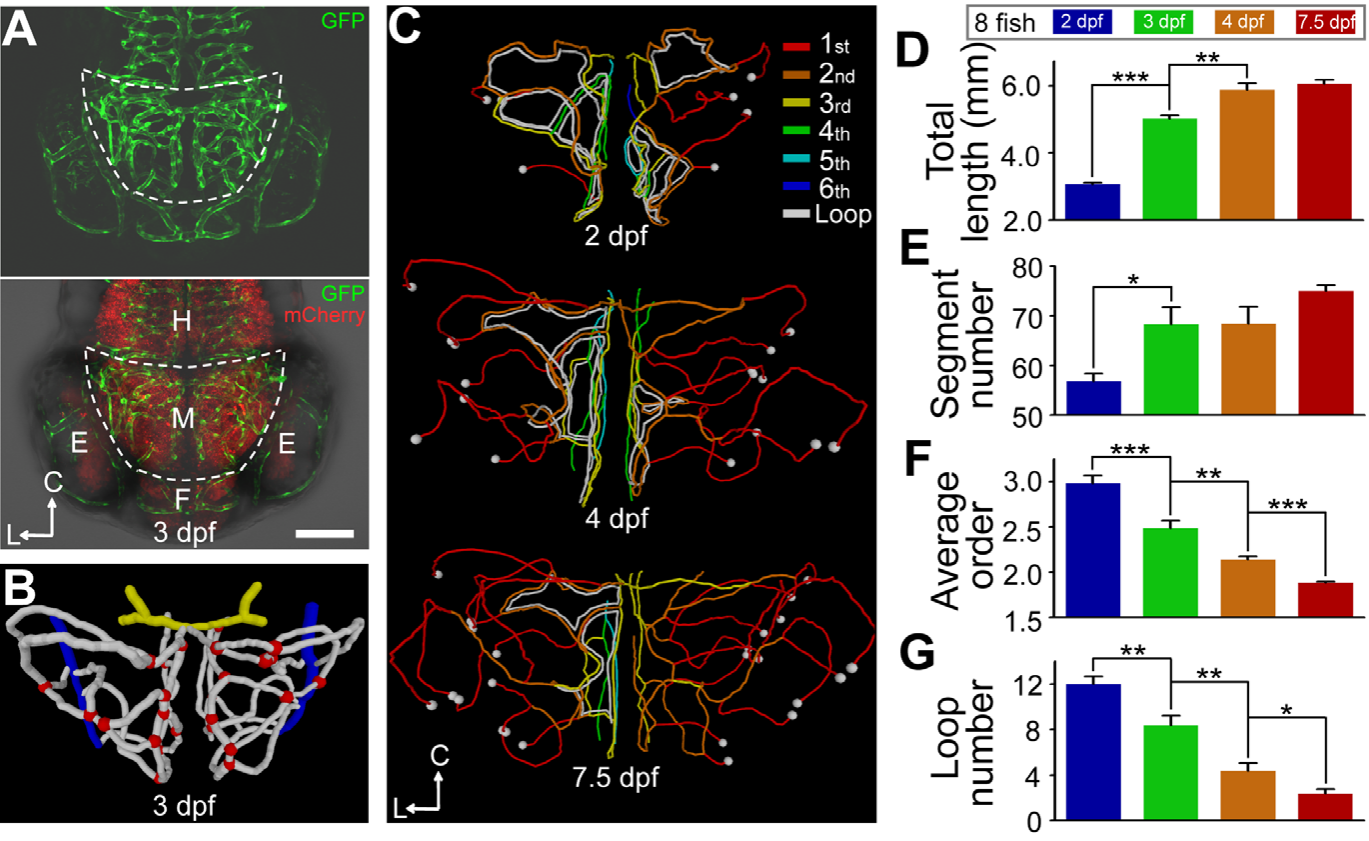
Structure changes of zebrafish midbrain vasculature during development. (A) Projected confocal images of a 3-dpf *Tg(kdrl:eGFP,HuC:gal4-uas-mCherry)* zebrafish larva showing the brain blood vasculature (green) and neural tissue (red, bottom). The dashed lines delineate the midbrain position. C, caudal; L, lateral; E, eye; F, forebrain; H, hindbrain; M, midbrain. Dorsal view, caudal is up. The same orientation is used for images and centerlines of whole-midbrain vasculature in all of the following figures. Scale, 100 µm. (B) 3-D reconstruction of the basal communicating artery (BCA, yellow), midbrain vasculature (white), and choroidal vascular plexus (CVP, blue) in the brain shown in (A). Red dots represent the branch points between vessel segments in the midbrain. (C–G) Developmental expansion and simplification of the midbrain vasculature. The data were obtained from eight larvae with each imaged at 2.0, 3.0, 4.0, and 7.5 dpf. (C) Representative midbrain vasculature centerlines of a larva at 2.0 (top), 4.0 (middle), and 7.5 dpf (bottom). Red, orange, yellow, green, cyan, and blue mark vessel segments with the 1^st^–6^th^ Strahler order, respectively. The white lines indicate internal vessel loops, and the white dots represent branch points between the CVP and midbrain vessel segments. (D–G) Summary of developmental changes in the total vessel length (D), segment number (E), weighted average segment Strahler order (F), and internal loop number (G) of the midbrain vasculature. * *p*<0.05; ** *p*<0.01; *** *p*<0.001 (paired Student's *t* test). Error bars, ± SEM.

To address how the brain vasculature develops, we first performed long-term serial confocal imaging of the midbrain vasculature in zebrafish larvae during 1.5–7.5 dpf. The brain vasculature was revealed by using the transgenic lines *Tg(kdrl:eGFP)* and *Tg(kdrl:RFP)*, which expressed GFP and RFP in ECs, respectively. The location of the midbrain was identified by using the double transgenic line *Tg(kdrl:eGFP,HuC:gal4-uas-mCherry)*, in which neurons expressed mCherry and the boundary between the midbrain and other brain areas could be clearly visualized (Figure 1A; Video S1). In the zebrafish, blood flow enters the midbrain via the basal communicating artery (BCA; Figure 1B, yellow) and exits the midbrain via the choroidal vascular plexus (CVP; Figure 1B, blue; see also [22]). We defined the midbrain vasculature as the vessel network between the BCA and CVP in the midbrain (Figure 1B, white).

The vessel ingression into the midbrain was initiated during 1.0–1.5 dpf. At 1.5 dpf, there were 5.2±0.2 angiogenic sprouts with a filopodium-like or expanded ending (Figure S1) and 4.2±1.0 vessel segments exhibiting blood flow (data from 10 larvae). At 2.0 dpf, a complex primitive vascular plexus with many circulatory loops (12.0±0.7) appeared (Figures 1C and S2). Accompanying the developmental expansion of the midbrain vasculature (Figures 1C and S2; Videos S2, S3, S4), both the total length and number of vessel segments markedly increased, with major changes occurring between 2.0 and 4.0 dpf (Figure 1D and 1E). These changes could be attributed to vessel elongation and new vessel addition through angiogenesis (Figure S3). Furthermore, using “segment Strahler order” and “internal loop” number to quantify the complexity of the vascular network (Figure S4A; see [23],[24]), we unexpectedly found that the weighted average order, internal loop number, and percentage of vessel segments located in internal loops all significantly decreased from 2.0 to 7.5 dpf (Figures 1F, 1G, and S5), with more than 70% of changes in these parameters occurring between 2.0 and 4.0 dpf. These findings indicate that the architecture of the midbrain vasculature undergoes substantial reduction of complexity after the initial formation of the primitive vascular network.

### Vessel Pruning Contributes to the Simplification of Developing Midbrain Vasculature

**Figure 2:**
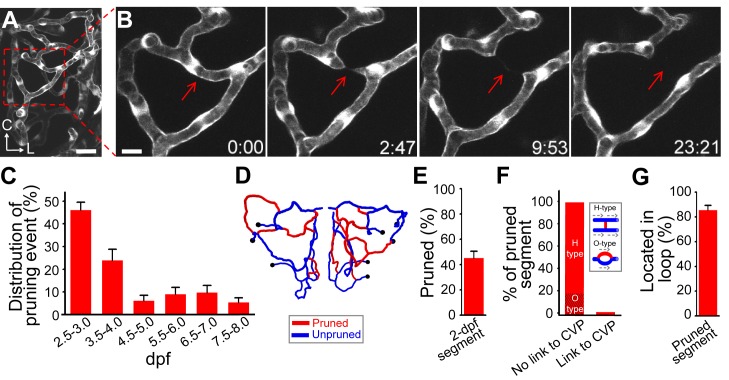
Occurrence of vessel pruning in the midbrain vasculature during development. (A) Projected confocal image of a left midbrain vasculature in a 3-dpf *Tg(kdrl:eGFP)* larva. (B) Serial images showing that a vessel segment (arrow) underwent pruning in the midbrain vasculature shown in (A, square). Time, hour:minute. Scales, 25 µm in (A) and 10 µm in (B). (C) Temporal distribution of vessel pruning events observed from eight larvae at each data point. (D and E) An example (D) and summary (E) of data showing that vessel segments formed at 2-dpf underwent extensive pruning during 2.0–7.0 dpf. The data in (E) were obtained from six larvae. (D) Centerline of a 2-dpf midbrain vasculature in which red and blue mark segments were pruned or unpruned during 2.0–7.0 dpf, respectively. The black dots represent branch points between the CVP and midbrain vessel segments. (F and G) Local structural features of pruned vessel segments. (F) Percentages of pruned segments that did not link to the CVP and were located in H-type (87/107) or O-type (19/107) of vascular microcircuits or directly linked to the CVP (1/107). The data were obtained from 18 larvae. Inset, schematic of H-type and O-type of vascular microcircuits. The dashed arrows in the inset indicate the direction of blood flow, and the red and blue lines represent pruned and unpruned vessel segments, respectively. (G) Percentage of pruned segments that were located in internal vessel loop. Error bars, ± SEM.

Angiogenesis and remodeling are the major processes of vascular development [7],[8]. Obviously, addition of new vessel segments via angiogenesis cannot explain the reduced complexity of the developing midbrain vasculature, especially the gradual disappearance of internal vessel loops. This prompted us to examine whether the developing vasculature in the zebrafish midbrain undergoes not only angiogenesis but also vessel pruning. We thus monitored the developmental process of each vessel segment in the midbrain vasculature of single larvae between 2.0 and 7.5 dpf and found that many early formed vessel segments were pruned during development. The process of vessel pruning began with gradual thinning, followed by complete collapse and eventual elimination of existing vessel segments (Figure 2A and 2B). About 70%±6% of pruning events occurred before 4.0 dpf (Figure 2C and red in Figure S2), and 45%±5% of segments formed at 2.0 dpf were eliminated by 7.0 dpf (Figure 2D and 2E). By analyzing the structural property of local vascular networks, we found that the vessel pruning was preferentially restricted to segments that were either located between two parallel primary vessels (87/107 events from 18 larvae; “H-type”) or were one of the two nearby segments that formed a small local loop (19/107; “O-type”; Figure 2F). For pruning occurring in “H-type” vascular microcircuits, the two vessel segments at each branch point of pruned segments always displayed the same blood flow direction (87/87), whereas for that occurring in “O-type” microcircuits, the pruned segment and its partner also showed same blood flow direction (19/19; Figure S6). The direction of blood flow was revealed by the movement of blood cells. Based on the analysis of blood flow direction in those microcircuits (Figure S6), pruned segments were not indispensable for local blood flow, suggesting that they are functionally redundant. Furthermore, among all pruned segments examined, only 1/107 linked directly to the CVP (Figure 2F), and 85%±4% of them (90/107) were located in internal vessel loops, including O-type local loops as well as large multi-segment loops (Figure 2G). These results indicate extensive pruning of loop-forming early vessel segments in the primitive vasculature.

**Figure 3:**
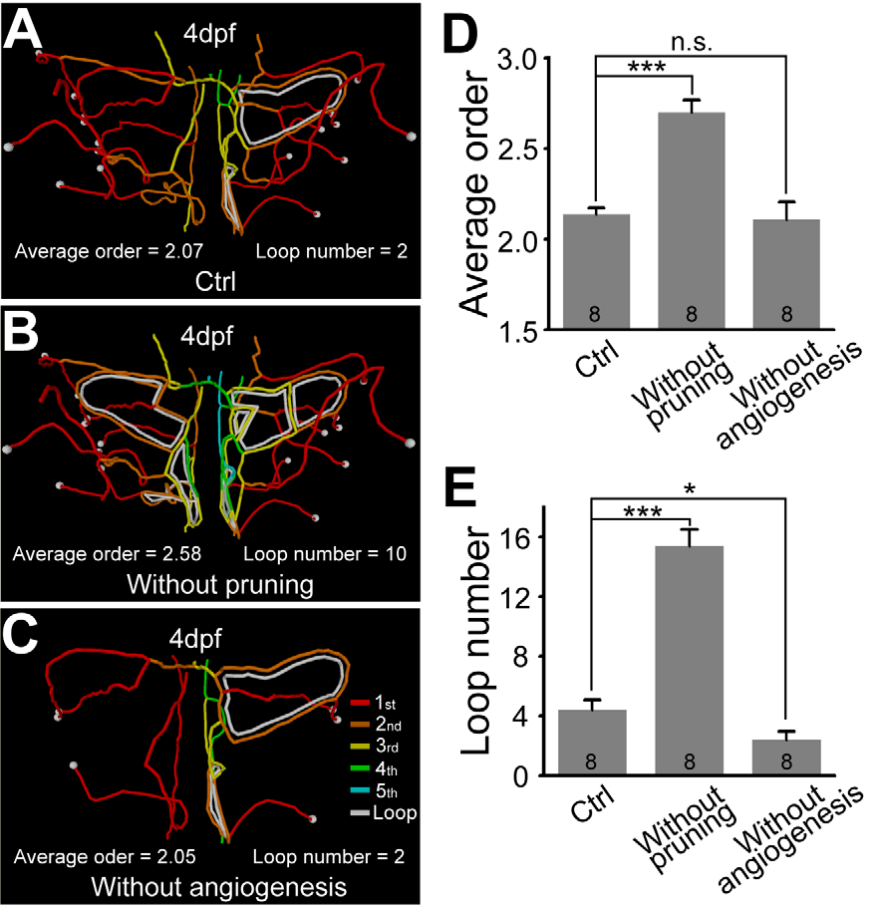
Vessel pruning reduces the complexity of the midbrain vasculature during development. (A–C) Representative centerline of 4-dpf midbrain vasculature under control condition (A), without pruning (B), or without angiogenesis (C). Red, orange, yellow, green, and cyan mark the vessel segments with the 1^st^–5^th^ Strahler order, respectively. The white lines indicate internal vessel loops, and the white dots represent branch points between the CVP and midbrain vessel segments. (D and E) Summary of the average segment Strahler order (D) and internal loop number (E) of 4-dpf midbrain vasculatures under control (“Ctrl”) condition or when artificially removing vessel pruning (“Without pruning”) or angiogenesis (“Without angiogenesis”) occurring between 2.0 and 4.0 dpf. The data were obtained from eight larvae. n.s., no significance; * *p*<0.05; *** *p*<0.001 (paired Student's *t* test). Error bars, ± SEM.

We next examined whether the vessel pruning is responsible for the developmental simplification of the midbrain vasculature, as indicated by the reduction of both the segment Strahler order and internal loop number. If all the pruned segments observed in a single fish during 2.0–4.0 dpf were artificially added to the vasculature observed for the same larva at 4.0 dpf to simulate the situation of “without pruning,” the vasculature would be much more complex than that observed, as indicated by both higher segment Strahler order and larger internal loop number (Figure 3A, 3B, 3D, and 3E; *p*<0.001, Student's *t* test; eight larvae). Consistently, in experiments to be described later, we found that reducing the occurrence of vessel pruning via pharmacological treatments did increase the Strahler order and internal loop number of the midbrain vasculature (see Figure S12). On the other hand, if all new vessels formed via angiogenesis between 2.0 and 4.0 dpf were artificially removed from the 4-dpf vasculature to simulate the situation of “without angiogenesis,” the vasculature complexity would be slightly decreased rather than increased (Figure 3A and 3C–3E). Thus, the vessel pruning rather than angiogenesis is responsible for the developmental simplification of the midbrain vasculature.

### Developmental Facilitation of Midbrain Blood Flow

**Figure 4:**
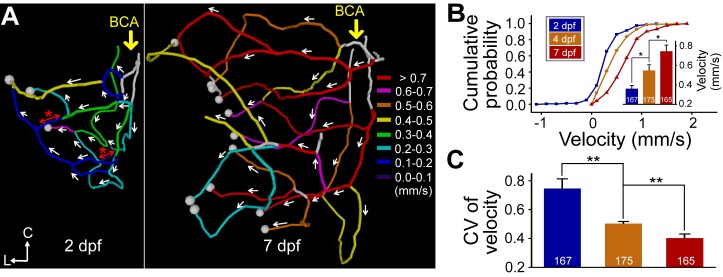
Developmental facilitation of the midbrain blood flow. (A) Representative spatial maps of blood flow velocity in a half midbrain vasculature of a larva at 2.0 dpf (left) and 7.0 dpf (right). The white arrows indicate the direction of blood flow in each vessel segment examined, and the red lines with an arrowhead at each end show bi-directional flow (asterisk). The large yellow arrows indicate the blood flow input from the BCA. The velocities were color-coded. The white lines indicate the segments to which line scanning was inaccessible (Materials and Methods). The white dots represent branch points between the CVP and midbrain vessel segments. (B and C) Summary of cumulative distribution (B) and variation (C) of flow velocity among different vessel segments in eight half midbrains of seven larvae with each imaged at 2.0, 4.0, and 7.0 dpf. The inset in (B) shows average values of flow velocities. The numbers on the bars represent the number of vessel segments examined. * *p*<0.05; ** *p*<0.01 (Student's *t* test). Error bars, ± SEM.

In principle, reducing loop number and segment order will optimize the efficiency of transport networks [24]. To examine the functional change accompanying the developmental simplification of the midbrain vasculature, we systematically monitored the blood flow velocity within the midbrain vasculature using *Tg(kdrl:eGFP,PU.1:gal4-uas-GFP)* double transgenic larvae, which expressed GFP in both vascular ECs and some unidentified blood cells. Blood flow velocity was calculated by measuring blood cell movement velocity with use of the axial line scanning and kymograph (Figure S7; see [25]) in all accessible vessel segments of single larvae at 2.0, 4.0, and 7.0 dpf. As shown by the examples of global velocity profile for a half midbrain of a larva measured at 2.0 and 7.0 dpf (Figure 4A), there was an overall increase in the flow velocity during development. By defining the flow towards the venous output CVP to be positive and the arterial input BCA to be negative, we found a significant increase in positive velocities as well as decreases in both negative velocities and coefficient of variation (CV) of velocities among different vessel segments from 2.0 to 7.0 dpf (Figure 4B and 4C; eight half midbrains from seven larvae). In addition, some vessel segments in 2-dpf but not 7-dpf midbrain vasculature exhibited bi-directional blood flow (Figure 4A, red asterisks). Interestingly, reducing the occurrence of vessel pruning via pharmacological treatments, which will be described later, could induce a slight decrease of the mean amplitude and a significant increase in the coefficient of variation (CV) of blood flow velocity in the midbrain (amplitude: 0.59±0.04 mm/s in control versus 0.51±0.03 mm/s in drug-treated, *p* = 0.153; CV: 0.49±0.04 in control versus 0.62±0.05 in drug-treated, *p* = 0.045; Figure S8). These findings indicate that the arteriovenous blood flow in the midbrain progressively becomes more efficient during vascular development, and vessel pruning may at least contribute to the formation of uniform global blood flow in the midbrain.

### Haemodynamic Changes Trigger Vessel Pruning

**Figure 5:**
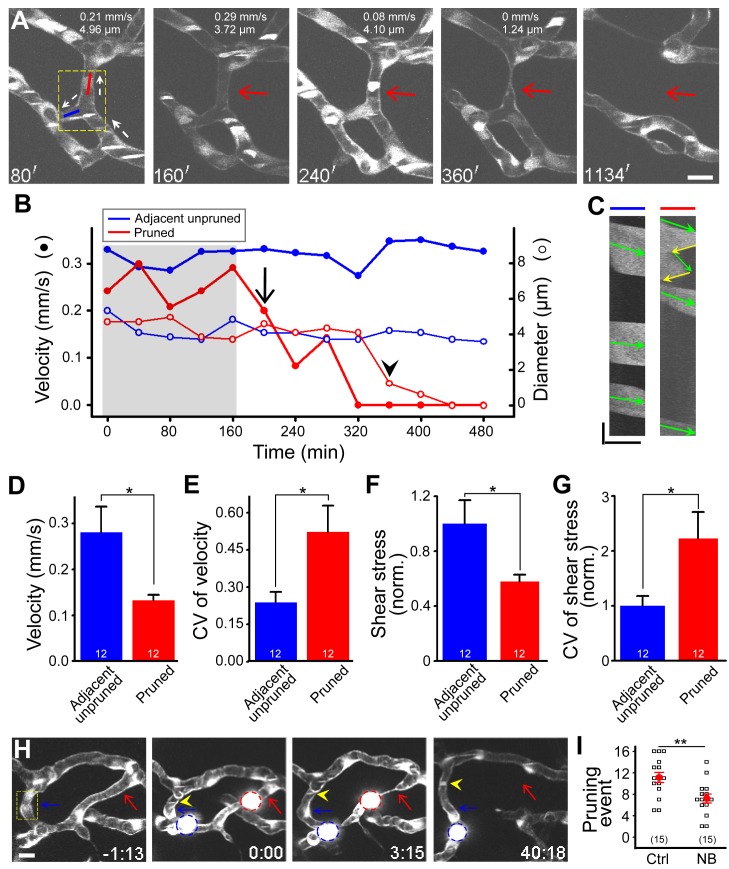
Changes in blood flow trigger vessel pruning. (A–G) Changes in blood flow before and during vessel pruning. (A–C) Data obtained from the same vessel segments. (A) Representative of simultaneous serial imaging and axial line scanning of midbrain vessels in a 2-dpf *Tg(kdrl:eGFP,PU.1:gal4-uas-GFP)* larva. Red and blue lines in the first panel indicate the site where axial line scanning was performed on a pruned (red arrow) and its adjacent unpruned segments, respectively. The numbers on the top of each panel represent the blood flow velocity and segment diameter of the pruned vessel segment. The white dashed arrows indicate blood flow direction. White signals in vessels were originated from moving blood cells that expressed GFP. The dashed square in the first panel marks the position from which blood flow is shown in Video S5. (B) Example showing changes with time of the blood flow velocity (filled circle) and diameter (open circle) of pruned (red) and adjacent unpruned (blue) segments before and during vessel pruning. The arrow and arrowhead mark the time point when the blood flow velocity showed an irreversible drop or the segment exhibited an obvious reduction in diameter, respectively. (C) Representative of kymographs showing bi-directional flow in the pruned segment (right) and uni-directional flow in an adjacent unpruned segment (left). The data were obtained from the pruned (red line) and unpruned (blue line) segments in (A) at the time point of 80 min. Green arrows indicate forward movement of blood cells, and yellow ones (right) indicate reverse flow in the pruned segment. (D and E) Average magnitude (D) and coefficient of variation (E) of flow velocities among different time points in pruned and adjacent unpruned segments before the time when an irreversible drop of flow velocity in pruned segments occurred (as indicated by the shadow region in B). The numbers on the bars represent the numbers of segments examined. (F and G) Normalized average magnitude (F) and coefficient of variation (G) of shear stress among different time points in pruned and adjacent unpruned segments before the time when an irreversible drop of flow velocity in pruned segments occurred (as indicated by the shadow region in B). (H and I) Effects of blood flow manipulation on vessel pruning. (H) Representative of serial imaging showing that the obstruction of blood flow by beads triggered vessel pruning. The beads were loaded several minutes before the time zero via the duct of Cuvier microinjection. The red and blue circles mark the beads that were successful or failed to block blood flow, respectively. The red and blue arrows mark the pruned and unpruned segments, respectively. The yellow arrowheads represent moving blood cells in the vessel segment in which a bead (blue circle) failed to block the blood flow. (I) Effects of norepinephrine bitartrate (NB) treatment at 2.0 dpf for 24 h on the occurrence of vessel pruning per larva. Each symbol represents data obtained from one larva. Scales, 10 µm in (A), 5.89 µm (*x*-axis) and 143 ms (*y*-axis) in (C) and 10 µm in (H). * *p*<0.05; ** *p*<0.01 (Student's *t* test). Error bars, ± SEM.

During the experiments of blood flow velocity mapping, we found that the average blood flow velocity in pruned segments before the initiation of pruning process (0.24±0.02 mm/s, *n* = 51) was significantly lower than that of unpruned segments (0.46±0.03 mm/s, *n* = 151, *p*<0.001), suggesting that changes in blood flow may play a role in triggering vessel pruning. We thus measured the blood flow velocities in both the pruned and its adjacent unpruned segments before and during the pruning process. By simultaneously measuring the changes in both the segment diameter and blood flow velocity, we found that in pruned segments, a significant irreversible reduction in the blood flow velocity before an obvious decrease in the segment diameter occurred in all 12 cases examined (Figure 5A and 5B for an example). The significant velocity reduction preceded the diameter decrease by 142±27 min (SEM). Furthermore, prior to the pruning onset, pruned segments frequently exhibited bi-directional blood flow during experiments (14/20; yellow arrows in Figure 5C right; Video S5). Compared to unpruned ones, pruned segments had a lower average blood flow velocity (Figure 5D; *p*<0.05) and a higher average CV for velocities at different times in each segment (Figure 5E; *p*<0.05). Consistently, the pruned vessel segment also showed a lower magnitude and higher variation of shear stress than its adjacent segment (Figure 5F and 5G; *p*<0.05). These findings imply that changes in blood flow may trigger vessel pruning.

To clarify the measurement of brain blood flow velocity, we then performed two lines of experiments. First, using double transgenic zebrafish *Tg(PU.1:gal4-uas-GFP,gata1:DsRed)*, in which DsRed are expressed in erythrocytes [26], we found that a majority of GFP-expressing cells in the circulation also expressed DsRed, though the number of GFP-expressing cells was less than that of DsRed-expressing cells (Figure S9A), indicating that some of GFP-expressing blood cells belong to a subset of DsRed-expressing erythrocytes. More importantly, there was no significant difference between the blood cell velocity measured by GFP or DsRed signals in midbrain vessel segments (0.68±0.07 versus 0.69±0.08 mm/s, *n* = 15, *p* = 0.44; Figure S9B and S9C). Second, we co-injected Fluosphere with green fluorescence (0.5 µm in diameter) and Dextran with red fluorescence (10,000 MW) into the circulation system of *Tg(kdrl:eGFP)* larvae to measure the speed of microspheres in midbrain vessel segments (Figure S10A). The microsphere speed can roughly reflect the velocity of blood plasma flow. Similar to the results obtained with blood cell velocity measured by GFP-expressing blood cells in *Tg(PU.1:gal4-uas-GFP)*, we found that pruned vessel segments displayed bi-directional plasma flow (Figure S10B) and exhibited a lower magnitude of plasma flow velocity and shear stress than adjacent unpruned vessel segments (Figure S10C and S10D).

Manipulation of brain blood flow was then performed to examine the causal relationship between blood flow changes and vessel pruning. When fluorescent micro-beads were loaded into blood circulation via microinjection at the duct of Cuvier, we found that vessel segments in which the blood flow was severely obstructed by the bead, as indicated by the failure of passage of blood cells, exhibited a reduction in their diameter and were eventually pruned in all cases examined (Figure 5H for an example, red arrow; *n* = 7). In contrast, when the bead failed to obstruct the blood flow, as indicated by the movement of blood cells (yellow arrowhead in Figure 5H; Video S6), no vessel pruning was observed (Figure 5H, blue arrow; *n* = 7). Conversely, increasing blood flow by incubating 2-dpf larvae in a medium containing norepinephrine bitartrate (NB, 60 µM; see [27]) for 24 h, a treatment that markedly elevated the heartbeat by 19%±1.5% (*p*<0.001) and brain blood flow velocity by 45%±16% (Figure S11; *p*<0.05) significantly reduced the frequency of vessel pruning (Figure 5I; *p*<0.01). In addition, the vasculature of NB-treated larvae exhibited higher Strahler order (*p*<0.05) and more internal loop number (Figure S12; *p*<0.05), as mentioned above. Furthermore, we reduced the heartbeat of zebrafish larvae by the treatment of MS222 (tricaine, 0.66 mg/ml; [28]) or 2,3-butanedione-2-monoxime (20 mM, BDM; [29]), or by morpholino (MO)-based down-regulation of *tnnt2* [30]. All these manipulations increased the occurrence of vessel collapse (Figure S13), which is an indicator of vessel pruning under various conditions we examined (see Figures 2B, 5A, 5H, 7B, 7C, 8A, and 8B).

**Figure 6:**
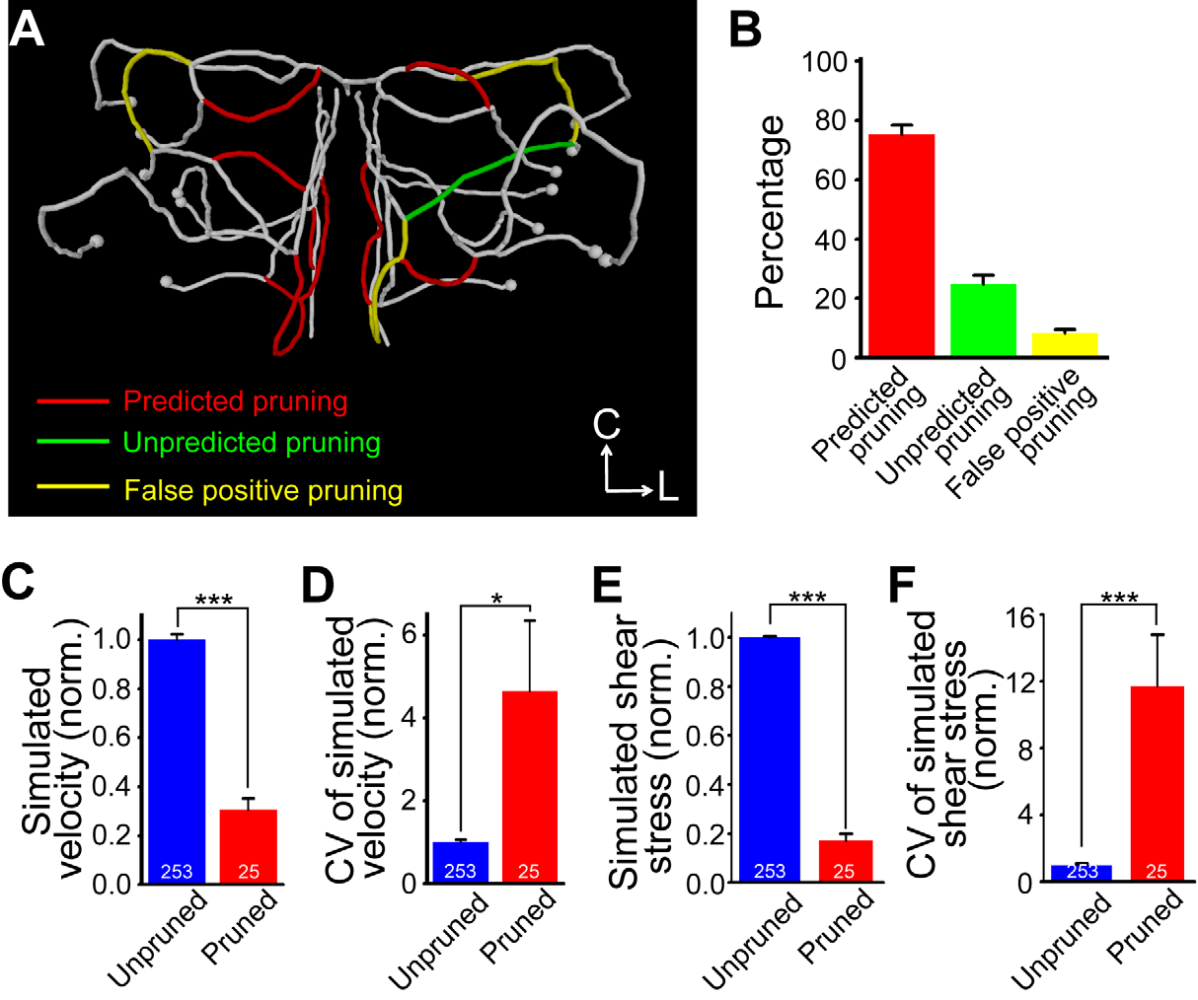
Prediction of vessel pruning by haemodynamics-based numerical simulation of vasculature refinement. (A) Representative centerline of a 3-dpf midbrain vasculature. Red, green and yellow mark vessel segments that were correctly predicted (“predicted pruning”), unpredicted (“unpredicted pruning”), or falsely predicted (“false positive pruning”) to be pruned, respectively. (B) Summary data obtained from seven simulated vasculature. (C and D) Average magnitude (C) and coefficient of variation (D) of simulated flow velocity in unpruned and pruned vessel segments. (E and F) Average magnitude (E) and coefficient of variation (F) of simulated shear stress in unpruned and pruned segments. The numbers on the bars represent the numbers of vessel segments simulated. * *p*<0.05; *** *p*<0.001 (Student's *t* test). Error bars, ± SEM.

Further numerical simulation of haemodynamic effects on vascular refinement was performed to predict which segments would be pruned in realistic zebrafish midbrain vasculature (see Materials and Methods). The prediction was then compared with observed results. Figure 6A depicts an example of simulation-predicted (“predicted pruning”+“false positive pruning”) and observed vessel pruning (“predicted pruning”+“unpredicted pruning”) in a 3-dpf midbrain vasculature. For seven realistic vasculatures simulated, 75%±3% of observed pruning events were predicted (red), and 8%±1% of segments were false-positively predicted as pruned segment (yellow, Figure 6B). Consistent with experimental observations (see Figure 5D–5G), predicted pruned segments prior to pruning exhibited much lower average magnitude and higher variation of both flow velocity (Figure 6C and 6D) and shear stress (Figure 6E and 6F) than predicted unpruned ones. Taken together, these results support the notion that changes in blood flow of vessel segments are responsible for triggering vessel pruning.

### Vessel Pruning Is Mainly Associated with Lateral Migration of Endothelial Cells

**Figure 7:**
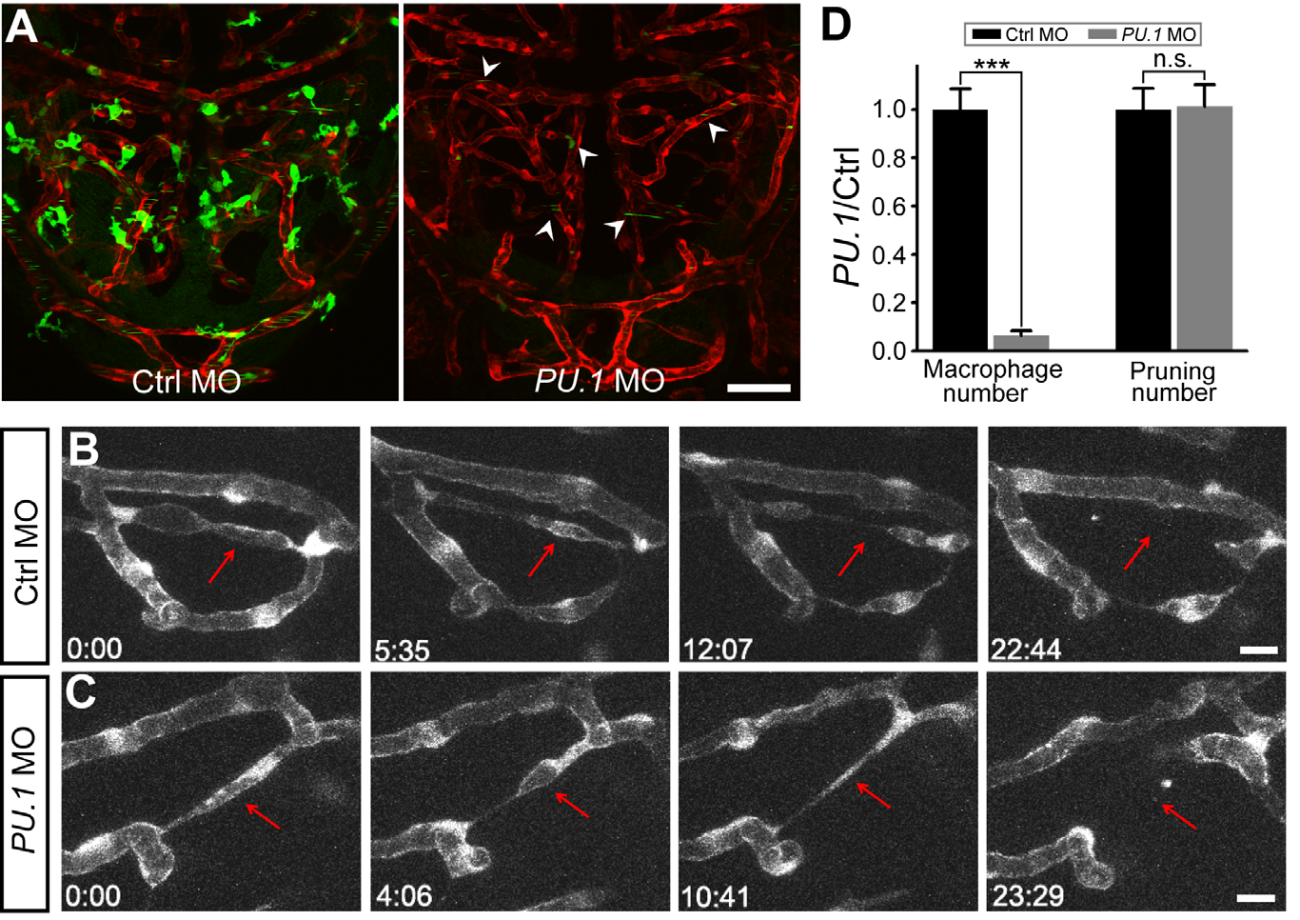
Macrophages are not required for vessel pruning. (A) Projected confocal images showing midbrain vasculature (red) and macrophages (green) in control MO- (left) and *PU.1* MO-injected (right) *Tg(kdrl:RFP,PU.1:gal4-uas-GFP)* larvae at 3 dpf. The green signals in the vessels (arrowheads) were originated from non-specific expression of GFP in blood cells. (B and C) Representative of serial images showing vessel pruning (arrows) in a control MO- (B) and *PU.1* MO-injected (C) larvae. (D) Summary of *PU.1* knockdown effects on macrophage development and vessel pruning occurrence in the zebrafish midbrain. The data were obtained from 16 larvae in each group. Scales, 50 µm in (A) and 10 µm in (B). n.s., no significance; *** *p*<0.001 (Student's *t* test). Error bars, ± SEM.

Previous studies of hyaloid vasculature, which is destined to be completely regressed during development, showed that vessel pruning is mediated by macrophage-dependent apoptosis of ECs [20],[21]. To examine whether EC apoptosis is involved in the vessel pruning, we performed TUNEL staining to detect apoptosis signal in the zebrafish midbrain during vascular development (Figure S14). In all 27 larvae at 3 dpf, only two ECs in the midbrain exhibited TUNEL-positive signal. As there were 11.1±0.9 events of vessel pruning occurring in the midbrain of each zebrafish larva at 3 dpf, it indicates that EC apoptosis is not a major cause of vessel pruning. We further investigated the role of macrophage in the midbrain vessel pruning by down-regulating the expression of *PU.1*, a transcription factor required for macrophage differentiation [31]. This was achieved by microinjection of *PU.1* morpholino oligonucleotide (MO) into 1–2-cell stage of *Tg(kdrl:RFP,PU.1:gal4-uas-GFP)* embryos, in which GFP was expressed by most of macrophages as well as a minority of blood cells [31]. In *PU.1* MO-injected embryos, macrophages were largely absent (Figure 7A and 7D), whereas the vessel pruning in the midbrain was not affected (Figure 7B–7D), suggesting that macrophages are not required for vessel pruning in the zebrafish midbrain vasculature. In addition, we found that VEGFA, Angiopoietin-2, and hypoxia are not important for vessel pruning. Morpholino-based downregulation of *VEGFA* and *Angiopoietin-2* did not significantly affect the pruning ratio of midbrain vessels (Figure S15). DMOG treatment, which is believed to induce hypoxic response in zebrafish larvae [32], also did not affect vessel pruning (Figure S16).

**Figure 8:**
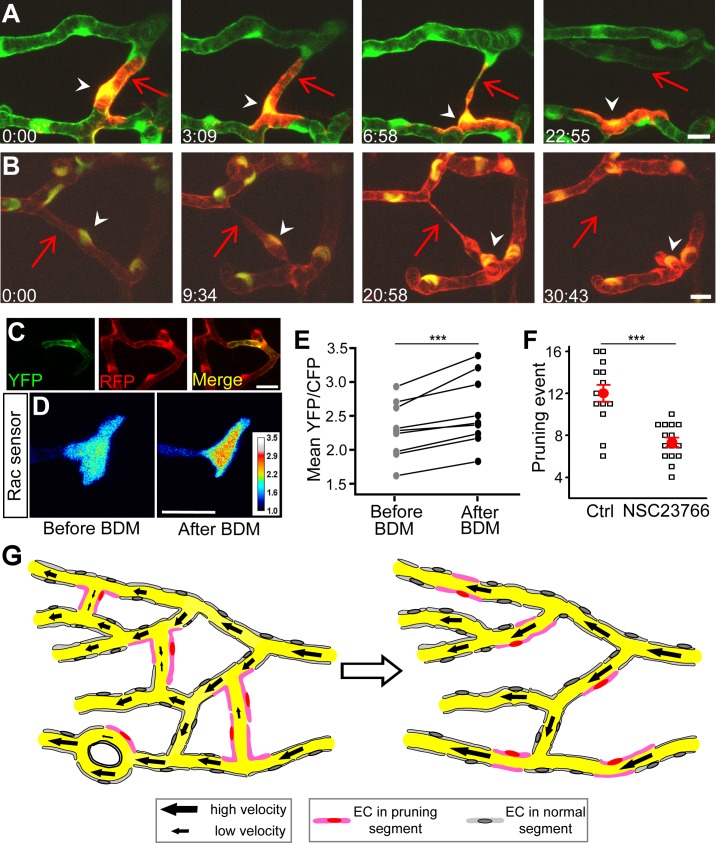
Vessel pruning is associated with endothelial cell migration and involves Rac1 activity. (A and B) Representative of tracing of single EC (A) or EC nuclei (B) showing that ECs (arrowheads) in pruned segments (arrows) migrated to adjacent unpruned segments during vessel pruning. The mCherry was mosaically expressed in single ECs of *Tg(kdrl:eGFP)* embryos (A), and *Tg(kdrl:RFP,fli1:nEGFP)* larvae were used to trace single EC nuclei (B, yellow). The arrows mark pruned vessel segments, and the arrowheads mark a migrating EC (A) or EC nucleus (B). Scales, 10 µm in (A and B). (C) Mosaic expression of the Raichu Rac1 FRET sensor in vascular endothelial cells of *Tg(kdrl:RFP)* zebrafish brain. YFP and RFP signals indicate FRET sensor and vascular endothelial cells, respectively. (D) Representative images showing the emission ratio (YFP/CFP) of the same EC expressing Rac1 FRET sensor before and after blood flow reduction induced by 30-min BDM treatment. The intensity of Rac1 FRET signal is color-coded. (E) Summary of data. Data obtained from the same EC are connected by a line. (F) Effect of NSC23766 treatment on the occurrence of vessel pruning per larva. NSC23766 was applied from 2 to 3 dpf and the pruning event was examined between 2 and 3 dpf. Each open symbol represents data obtained from one larva, and the red ones represent the mean values. (G) Working model. In the primitive vasculature (left), some vessel segments exhibit low and unstable blood flow, and ECs located at these segments (pink) undergo lateral migration, leading to vessel pruning. This pruning consequently results in the formation of a simplified vasculature with reduced numbers of internal vessel loop and segment Strahler order (right). Scales, 20 µm in (C) and (D). *** *p*<0.001 (paired Student's *t* test in E and unpaired Student's *t* test in F). Error bars, ± SEM.

To further investigate the cellular mechanism of vessel pruning, we traced the fate of ECs during the pruning process by mosaically expressing mCherry in single ECs of *Tg(kdrl:eGFP)* embryos. We found that ECs located in pruned segments did not undergo apoptosis but migrated to adjacent unpruned segments after vessel elimination in all cases examined (5/5; see Figure 8A for an example). Tracing EC nuclei in *Tg(kdrl:RFP,fli1:nEGFP)* larvae, in which GFP was localized in the nucleus of ECs [33],[34], also showed that at least 82% of EC nuclei associated with pruned segments migrated to adjacent unpruned segments (37/45; see Figure 8B for an example). To understand how haemodynamics induce the migration of ECs, we next examined whether Rac1 activity in ECs could be regulated by changes in blood flow. We used a Raichu FRET sensor to detect Rac1 activity in ECs in intact zebrafish larvae. This Rac1 FRET sensor has been described and verified in previous studies [35]–[37]. Rac1 FRET sensor was mosaically expressed in single brain ECs by injecting Kdrl-Rac1-FRET plasmid into *Tg(kdrl:RFP)* zebrafish eggs (Figure 8C). We found that the reduction of brain blood flow by BDM treatment significantly increased Rac1 activity in midbrain ECs (Figure 8D and 8E), suggesting that Rac1 activity in ECs is downstream of haemodynamics. Furthermore, the blockade of Rac1 activity by the treatment of the Rac1 inhibitor NSC23766 [38] significantly impeded the occurrence of vessel pruning in the zebrafish midbrain (Figure 8F). Considering that Rac1 is important for cell migration [39], our data suggest that the reduction of brain blood flow in pruned vessel segments activates Rac1 in ECs, leading to EC migration-associated vessel pruning.

## Discussion

Our findings demonstrate the existence of vessel pruning and its crucial role in the formation of the midbrain vasculature (Figure 8G). This vessel pruning leads to a developmental reduction of vascular network complexity that may facilitate efficient routing of midbrain blood flow. We further revealed that changes of blood flow are responsible for triggering vessel pruning. Furthermore, we showed that vessel pruning is mainly associated with migration of ECs from pruned to adjacent unpruned segments.

### Roles of Vessel Pruning in the Formation of Brain Vasculature

Refinement is believed to be a general process for the formation of complex biological systems [40]. During development, vasculature refinement has been reported in the hyaloid, retina, and yolk sac [20],[21],[41]–[44]. We here found that the developing brain vasculature also undergoes extensively refinement mainly through vessel pruning. Because it preferentially occurs at loop-forming early vessel segments, which usually exhibit inefficient blood circulation and are functionally redundant, the vessel pruning leads to the simplification of developing brain vasculature with improved blood flow.

The formation of zebrafish midbrain vasculature does not seem to follow strictly a pre-determined pattern as that observed in the zebrafish trunk (see [33],[34]). In the zebrafish midbrain, the primitive vascular plexus exhibits high complexity and is largely variable among different larvae, and the formation of the relative mature vasculature pattern requires substantial refinement processes to simplify the vasculature during development. The present study identified vessel pruning as an important process for vasculature refinement. First, we found that the vessel pruning in the midbrain mainly occurs at early formed segments, many of which form loop structures (Figure 2). On the other hand, the majority of later-formed vessel segments via sprouting angiogenesis from the early formed plexus do not undergo pruning and directly contribute to the structure of mature vasculature. Importantly, the vasculature complexity is not obviously reduced by angiogenesis (Figure 3). Second, the relatively mature midbrain vasculature at 7.5 dpf is hierarchical, with a low average Strahler order and fewer vessel loops (Figure 1). This hierarchical vasculature becomes apparent by 4 dpf, the time point when most of vessel pruning events have already occurred (Figure 2). Third, reducing the occurrence of vessel pruning increases the Strahler order and internal loop number of midbrain vasculature (Figure S12).

A central question in vascular development is how genetic and epigenetic factors determine the final topological structure of vasculature. Based on the present study, we speculate that the process of midbrain vasculature formation represents a developmental strategy for establishing an efficient vessel network via blood flow-mediated refinement, reminiscent of activity-dependent refinement of developing neural circuits [40]. Angiogenesis is initiated when the vasculature cannot meet the demand for blood supply [45], whereas vessel pruning eliminates those vessel segments that are functionally redundant or obstructive. By using these two cellular processes, the vasculature is shaped to an architecture that meets the tissue requirement for blood flow.

### Vessel Pruning and Haemodynamic Changes

Haemodynamic force is reported to regulate vascular remodeling and arteriovenous differentiation in the yolk sac [43],[44],[46]–[48], remodeling and angiogenesis of the aortic arch [28],[49], formation of hindbrain arteriovenous connections [50],[51] (but see [52]), and development of hematopoietic stem cells [27]. Although previous studies have demonstrated the essential role of haemodynamic force for cardiovascular development [48],[53],[54], it remains unclear to what extent and the mechanism of how blood flow contributes to the refinement of complex vasculatures [46]. Our study provides evidence showing that changes in blood flow refine the complex interconnected vessel network to a simplified hierarchical structure with more efficient flow routing via triggering vessel pruning in the zebrafish midbrain (Figure 8G). This haemodynamics-based vessel pruning may be a universal post-angiogenic mechanism for refinement of developing vasculatures in other organs.

In previous studies concerning haemodynamic effects on vasculature development, manipulations of blood flow in whole embryos were usually applied [27],[28],[44],[46], and the temporal sequence between changes in haemodynamics and vessel morphology has not been systematically examined. By simultaneously monitoring changes in both local blood flow and vessel morphology, we found that prior to the pruning, the pruned segment displays lower blood flow velocity, high variability (CV) of the flow velocity, and high probability of bidirectional blood flow (Figure 5). Either the reduced level of flow velocity or the change in the flow velocity, or both, could be the cause for vessel pruning. Due to technique limitation, we cannot discriminate these possibilities now. Furthermore, in our study, we microinjected fluorescent micro-beads to locally manipulate blood flow at single vessel segments. This experiment explicitly indicates that local changes in haemodynamic force are sufficient for triggering vessel pruning. The haemodynamics dependency of vessel pruning is further confirmed by our numerical simulation, which was mainly based on the assumption that shear stress determines the change of vessel segment radius [55]. Although the real vasculature is quite complex, the fate of each vessel segment can largely be predicted.

### Difference of Vessel Pruning between Early and Later-Formed Vessel Segments

In this study, we found that the vessel segments that directly connect to the CVP are rarely pruned. This may be due to the relative stable haemodynamics in those CVP-connecting vessel segments and their important role in directly draining midbrain blood into the main vein CVP. In our experiments, bi-directional blood flow was never observed in those segments. During early development between 1.5 and 2.0 dpf, many angiogenic sprouts in the midbrain are not originated from the CVP but from the midbrain vasculature (green arrows in Figure S1), leading to the formation of an early vasculature with a minority of vessel segments connecting to the CVP (12.2%±0.8%) and many vessel segments locating in loops (70.0%±2.1%). The blood flow in some of those CVP-unconnecting and/or loop-forming vessel segments is quite unstable. Thus, the early formed vessel segments are more prone to be pruned. However, angiogenic sprouts at late stages are mostly originated from the CVP (see Figure S2). Those later-formed vessel segments make a direct connection between the midbrain vasculature and the blood flow exit CVP. This may explain why the early formed but not later-formed vessel segments are preferentially pruned.

As angiogenesis and vessel pruning are interwoven and we currently have no way to selectively manipulate angiogenesis without effect on vessel pruning and vice versa, it is a challenge to examine the relationship between them at present. During our experiments, we found that some of newly formed vessel segments connected to existing segments not at their branch points but at the midway and thus divided the existing ones into two parts, leading to marked shunt of local blood flow (unpublished data). One part of the divided segments was finally pruned in some cases (*n* = 6). Thus, it seems that angiogenesis may affect vessel pruning by changing local blood flow.

### Migration of Endothelial Cells Associates with Brain Vessel Pruning

Regression of blood vessels has been intensively examined in hyaloids [20],[21] and was also implicated in retinae [41],[56]. Unlike the global vessel elimination observed in hyaloid vascular plexus [20], vessel pruning in the zebrafish brain is selectively restricted to loop-forming early vessel segments. Macrophage-induced apoptosis of ECs is responsible for vessel elimination in hyaloids [20]. Interestingly, in the present study, we found that macrophage is not required for vessel pruning in the zebrafish midbrain. Together with the evidence that TUNEL-positive apoptosis signal does not co-localize well with regressed vessels in retinae [42], it suggests the involvement of alternative mechanisms underlying vessel pruning in the brain.

By tracing the dynamic behavior of single ECs, we demonstrated for the first time, to our knowledge, that vessel pruning is highly associated with lateral migration of ECs. As the brain by itself does not generate ECs [5],[6], such migration rather than apoptosis may represent an efficient way for reconstructing the brain vasculature [57]. Considering that 8 of 45 EC nuclei were lost during our tracing experiments, we cannot exclude the possibility that the apoptosis of ECs is also involved, even though we did not observe TUNEL signals associated with zebrafish midbrain ECs. During vessel pruning, migrating ECs in pruned segments display highly dynamic changes in their morphology (Figure 8A), including elongation and migration of their cell bodies, and segregation with one of the nearby ECs that most likely involves the disruption of EC-EC junctions [58]. Furthermore, blood flow-associated mechanical forces can affect many EC functions, including gene expression, proliferation, apoptosis, as well as migration [46],[48],[53],[54]. Previous work demonstrated that Rac1 activity in cultured ECs can be transiently increased by up-regulation of blood flow [59], suggesting that Rac1 may respond to changes in blood flow. Our study provides another line of evidence for supporting this notion. A reduction of blood flow may also trigger other intracellular signals responsible for EC migration [53],[54] toward adjacent segments that we observed during brain vessel pruning and supposed disruption of EC-EC junctions. We believe that vessel pruning may require coordination of more than one signaling pathway downstream to blood flow.

### Conclusion

Using interdisciplinary approaches, we found that blood flow changes trigger vessel pruning via EC migration, leading to simplification of early formed complex vasculature. This study not only provides a novel cellular mechanism for vessel pruning but also sheds light on understanding how haemodynamic forces shape the formation of vascular architecture during development [46].

## Materials and Methods

### Zebrafish Husbandry

Adult zebrafish (*Danio rerio*) were maintained in the National Zebrafish Resources of China (NZRC, Shanghai, China) with an automatic fish housing system (ESEN, Beijing, China) at 28°C following standard protocols [34]. The transgenic lines of *Tg(kdrl:eGFP)^s843^* [60], *Tg(kdrl:RFP)^la4^* [61], *Tg(fli1:nEGFP)^y7^* [62], *Tg(gata1:DsRed)^sd2^* [26], *Tg(PU.1:gal4-uas-GFP)^zf149^* [31], and *Tg(HuC:gal4-uas-mCherry)* [63] were described previously. Embryos were raised under a 14 h∶10 h light∶dark cycle in 10% Hank's solution, which consisted of (in mM): 140 NaCl, 5.4 KCl, 0.25 Na_2_HPO_4_, 0.44 KH_2_PO_4_, 1.3 CaCl_2_, 1.0 MgSO_4_, and 4.2 NaHCO_3_ (pH 7.2), and were treated with 0.003% 1-phenyl-2-thiourea (PTU, Sigma) to prevent pigment formation. Due to manual removal of chorion at 1 dpf for long-term serial imaging [64], the developmental stages of larvae we used was a little earlier than that previously reported [22]. The handling procedures were approved by Institute of Neuroscience, Chinese Academy of Sciences.

### In Vivo Confocal Imaging

Imaging was performed on 1.5–8.0 days post-fertilization (dpf) zebrafish larvae at room temperature (26–28°C). Larvae were embedded in 1% low-melt agarose (Sigma) for imaging without anesthetic and were dug out for husbandry if subsequent imaging of the same larva was performed after >20 min. Developmental changes in whole-midbrain vasculature morphology were serial imaged from the same larva at different developmental stages with intervals of 40 min to 24 h. To trace the fate of each vessel segment during development, long-term serial imaging of brain vasculature of the same larvae was performed. To confirm that the same vessel segments were traced and analyzed, we always compared the 3-D structures of midbrain vasculature obtained at adjacent time points (Figure S17). Considering that the processes of sprouting angiogenesis and vessel pruning in the zebrafish brain were quite slow, the difference between the 3-D structures of vasculature at adjacent time points was not dramatic, and each vessel segment could be reliably traced. To calculate the “pruning number,” we always imaged the same larvae for at least two time points. If a vessel segment displays lumenized morphology at the first time point but exhibits a collapsed shape or disappeared completely at the second time point, we will count it as a pruned event.

Imaging was carried out with an Olympus Fluoview 1000 confocal microscope (Tokyo, Japan). Lumplfl 40× (W/IR; NA, 0.80) and XLumplfl 20× (W/IR; NA, 0.95) objective lenses (Olympus) were used. The *z*-step of imaging ranged from 1 to 3 µm. During experiments, a heating plate (CU-201, Live Cell Instrument) was always used to keep the temperature of imaging chamber solution at 28°C. Kdrl:mCherry was microinjected into *Tg(kdrl:eGFP)* embryos at one- or two-cell stage to mosaically label single vascular endothelial cells (ECs). Kdrl: Rac1-FRET-A422 was microinjected into *Tg(kdrl:RFP)* embryos at one- or two-cell stage to detect Rac1 activity in vascular endothelial cells (ECs) as previously described [35],[36]. FRET imaging was performed with a Fluoview 1000 confocal microscope using CFP/YFP/FRET mode. FRET signal analysis followed a previous protocol [35].

### Quantitative Analysis of Brain Vasculature Morphology

A computer-assisted method was developed to extract the skeleton of 3-D vasculature and quantitatively analyze its geometrical and topological properties, including 3-D coordinates (X-Y-Z) of points on the segmented vessel, segment number, segment length, segment diameter, internal loop number, and segment Strahler order number. The detailed processes were described below.

First, the raw image of vasculature was processed with a computational method to extract its 3-D skeleton. It consisted of three main sequential steps, including pre-processing, segmentation, and skeletonization. In pre-processing, 3-D deconvolution was firstly carried out to reduce convolution effects produced by microscope optics. Spatial isotropic *z*-stack images were then obtained with a linear image interpolation, and midbrain vasculature was localized according to 3-D brain structure images. After pre-processing, a progressive thresholding method was performed to segment vascular images, followed with 2-D region growth and 3-D morphological closing operation to yield solid vessels for subsequent skeletonization. The skeleton of midbrain vasculature was extracted based on iterative morphological thinning and judgment of connectivity maintenance. The 3-D structure of brain vasculature was reconstructed using Neurolucida software (MBF Bioscience, Williston, VT).

With computer-assisted algorithms, segmented vessels and their skeleton of the midbrain vasculature were then used for automatic quantitative analysis of vasculature geometrical and topological properties, including segment length, segment number, segment diameter, segment Strahler order, and internal loop. A vessel segment was defined as a region between two adjacent vessel branch points. Each segment's length was calculated as the sum of 3-D Euclidean distance between two adjacent points along each segment skeleton. At each point of the skeleton, segment diameter was computed through 3-D dilation operation with one sphere element, whose diameter was increased stepwise. The dilation would not stop until the increasing sphere reached the boundary of segmented vessels. The diameters of pruned and adjacent unpruned segments were calculated as the minimum value of the average diameter of each of five successive points on the segment. If the skeleton of the pruned segment was disconnected, the segment diameter was assigned as zero.

Both “segment order” and “internal loop” were used to characterize the complexity of midbrain vascular network (Figure S4). The higher percentage of high order segments and more internal loops indicate a more complex network [23],[24]. The “segment order” was analyzed by the Strahler ordering method [23], which follows three rules: (1) If the segment is a leaf, its Strahler order number is one; (2) if the segment has one child segment with Strahler number *i* and all other child segments have Strahler numbers less than *i*, then the Strahler number of the segment is *i* again; (3) if the segment has two or more children with Strahler number *i* and no child segments with a greater number, then the Strahler number of the segment is *i+1*. In our system, vessel segments directly connecting with the CVP were assigned as the first order or “leaf” segment. Beginning from the first order vessels, the other segments along the 3-D skeleton from the CVP to the BCA were orderly assigned with sequential order numbers (second, third, fourth, and so on) automatically. The average order of the midbrain vasculature in each larva was calculated as the weighted arithmetic average, which was the sum of the percentage of segments at each order weighted by its corresponding order number.

The “internal loop” was defined as the non-overlapping recurrent connections with a minimal number of segments. We traced the 3-D vasculature skeleton starting from a branch point. If we could return to the starting branch point, it meant that there was a vessel loop. Then, we used the following rules to precisely define and count vessel loops. First, the smallest loop consisting of minimal segment numbers was chosen. Second, only non-overlapping loops were counted.

### Measurement and Calculation of Blood Flow Velocity

Blood cells were mosaically labeled by GFP in *Tg(PU.1:gal4-uas-GFP)* or DsRed in *Tg(gata1:DsRed)* embryos. The blood flow velocity in vessel segments was measured by monitoring the movement of GFP- or DsRed-expressing blood cells with the axial line scanning (ALS) method as previously reported [25],[65]. For globally mapping the developmental changes in blood flow, blood cell velocities among vessel segments in the same larvae at 2.0, 4.0, and 7.0 dpf were successively measured. Except vessel segments with centerline perpendicular to scanning plane, a situation preventing us from carrying out line scanning, all other accessible midbrain vessel segments in single larvae were scanned with 800–850 Hz for more than 15,000 times per segment at each time point. The length of scanned lines was 9.5±0.3 µm. The flow velocity of each segment was averaged from 100±16 blood cells. The variation of velocity among different vessel segments was calculated. To examine the temporal profile of blood flow velocity in both pruned and adjacent unpruned segments, *Tg(kdrl:eGFP,PU.1:gal4-uas-GFP)* larvae were used to monitor both morphology and blood flow velocity in pruned and adjacent unpruned segments before and during vessel pruning. Velocity measurement and morphology imaging were performed with a 40-min interval and repeated until vessel pruning occurred. To measure the velocity of blood plasma flow, we co-injected Fluosphere with green fluorescence (0.5 µm in diameter; Invitrogen) and Dextran with red fluorescence (10,000 MW) into the circulation system of *Tg(kdrl:eGFP)* larvae to measure the speed of microspheres in vessel segments. The microsphere speed can roughly reflect the velocity of blood plasma flow.

To calculate the blood cell velocity, kymographs were analyzed by an automated method. Kymograph was obtained from repeated ALS. It can provide both the velocity and direction of blood flow in scanned segments [25]. A user-defined threshold was firstly set to segment blood cells. After noise removal, all the connected regions of each segmented blood cell were extracted. The coordinates of intersection points between each connected region and its image boundaries were then obtained. The intersection points at the left and right boundaries with minimum, average, and maximum coordinates were linked to each other for calculating the starting, average, and ending velocities of each blood cell, respectively (Figure S7). For the uni-directional blood flow, the average velocity was used, whereas for the occasional bi-directional blood flow (Figure 5C right), the starting and ending velocities were used for forward and backward directions, respectively. The same approach was used to calculate the velocity of Fluosphere. The coefficient of variation (CV) of blood cell velocity among different time points was used to estimate the variation of velocity in each vessel segment. Considering that the velocity dropped in pruned vessels even before the initiation of pruning process, we only took the velocities before the velocity drop into CV calculation, as indicated by the shadow region in Figure 5B. The same method was applied for measuring the average velocity and associated CV in numerical simulation data.

### Shear Stress Calculation

Based on the equation of 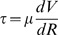 [51],[66], we calculated the mean and coefficient of variation (CV) of relative shear stress (τ) in pruned and adjacent unpruned vessel segments. Blood cell velocity (V) was measured as described above, and the radius (R) of vessel segments was calculated based on their 3-D morphology.

### Haemodynamics-Based Vasculature Refinement Model for Predicting Vessel Pruning

First, each vessel segment in the midbrain vasculature was traced during development by serial imaging of 3-D midbrain vasculature of the same larvae at different stages (see Figure S2). Then, we quantitatively analyzed the geometrical and topological properties of the vasculature by using self-written software. The 3-D coordinates (X-Y-Z) of each branch point of vessel segments were then obtained. We assigned each vessel segment a number and located each segment by the X-Y-Z coordinates of its two branch points. The length and radius of each vessel segment were also obtained. Based on these parameters, we re-constructed the 3-D structure of midbrain vasculature by using Matlab. In Matlab, a refinement model of vasculature according to haemodynamics [55],[67] was set up to predict the pruning segments based on the topological and geometrical information of 3-dpf zebrafish midbrain vasculature. According to Poiseuille's law [68],[69], we defined the resistance of vessels (*R*_ei_) and calculated the shear stress by:


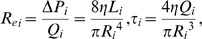


where *R_i_*, *L_i_*, *Q_i_*, *τ_i_*, and *ΔP_i_* were the radius, length, blood flow, shear stress, and pressure drop of the i^th^ segment, respectively. During haemodynamics-based adaptation, the length of each segment was assumed to be fixed. The change in vessel radius with time was assumed to depend on the shear stress:


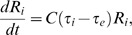


where *C* was a constant and *τ*_e_ was the preset constant shear stress. The pressure was assumed to be uniform at all the inlets of the vessel network, and the shear stress was fixed at the preset value *τ*_e_ in all the outlet vessels. Therefore, the blood flow was fixed in these vessels, and the radius of these vessels did not change. At each time step, we used the radius and lengths to calculate the resistances, then solved for the blood flows and blood pressure at all vessel junctions by an analogy to the electric circuit. Then we calculated the shear stresses and used them to adapt vessel radius. When the radius of a vessel segment became smaller than 0.1 µm, the vessel was regarded as a pruned one and its radius was fixed afterwards to avoid numerical problems. We started from the 3-dpf vasculature of zebrafish midbrain and adapted the system until the steady state was reached through 5,000 time steps. Thus, the changes with time of the radius, shear stress, and blood flow velocity for each segment were obtained, and the pruning fate of each segment was predicted. Finally, we compared the fate of each vessel segment in the mathematical model with that observed experimentally to quantitatively examine the degree to which the model can predict pruned segment: (1) If they were matched, we defined it as “predicted pruning”; (2) if the pruning of segments was predicted in the simulation but was not observed during experiment, we defined it as “false positive pruning”; (3) if the pruning of segment was observed in the experiment but was not predicted by the simulation, we defined it as “unpredicted pruning.” In addition, we also compared the parameters (magnitude and CV of both blood flow velocity and shear stress) calculated by the experiments and numerical simulations in pruned segments with those in the unpruned segments and examined whether the mathematical model can fit the experimental data.

To examine the effect of blood flow pulsatility on the numerical simulation, we further performed simulation when a fluctuating or constant blood flow with the same mean speed was inputted into the mathematical model (Figure S18A). Regardless of whether the blood flow was fluctuating or constant, the simulation results were not significantly different (Figure S18B and S18C). Although the time step required for vessel pruning completion was slightly different between two simulations (Figure S18C), the predicted pruning fate of vessel segments was almost the same, as indicated by the data that the radius of same vessel segments during the two simulations will finally approach zero (bottom panels in Figure S18B).

### TUNEL Staining

The brain of *Tg(kdrl:eGFP)* larvae at 3 dpf was coronally sliced and TUNEL staining was performed following the manufacturer's protocol (In Situ Cell Death Detection Kit TMR red, Roche; [70]). To confirm the TUNEL labeling, DNase I treatment was used as a positive control (Figure S14A), while labeling solution without terminal transferase in kit was used to replace TUNEL reaction mixture as a negative control (Figure S14B).

### Morpholino Oligo-Based Knockdown and Microinjection

Morpholino oligos (MOs) were purchased from Gene Tools (Philomath, OR). Lyophilized MOs were dissolved in nuclease-free water. The *PU.1* MO (5′-GATATACTGATACTCCATTGGTGGT-3′) [31], *Tnnt2* MO (5′- CATGTTTGCTCTGATCTGACACGCA-3′) [30], *VEGFA* MO (5′-GTATCAAATAAACAACCAAGTTCAT-3′) [71], *Angiopoietin-2* MO (5′-TCATTTGATCAGCCTCACCTGCGTC-3′) [72], or control MO (5′-CCTCTTACCTCAGTTACAATTTATA-3′) was pressure-injected into one-cell stage embryos with doses indicated in the figure legends.

### Reagents

Fluoresbrite beads (6.3 µm in diameter, 18141-2, Polysciences) and Dextran Alexa Fluor 568 (10,000 MW, D22912, Invitrogen) were loaded into blood circulation via the duct of Cuvier microinjection to randomly occlude brain vessel segments or label blood plasma, respectively. FluoSpheres carboxylate-modified microspheres (0.5 µm in diameter, green fluorescent) and Dextran Alexa Fluor 568 were co-injected into the circulation system for measuring the velocity of plasma flow. To enhance heartbeat and brain blood flow, 2-dpf larvae were treated with norepinephrine bitartrate (NB, 60 µM; Sigma) for 24 h [27]. The treatment of 2,3-Butanedione-2-monoxime (BDM, 20 mM; Sigma) and MS222 (tricaine, 0.66 mg/ml; Sigma) was performed following previous protocols [28],[29]. Larvae at 2 dpf were treated with NSC23766 (150 µM, Calbiochem) to decrease Rac1 activity [38]. Anti-Zebrafish VEGF antibody (1∶500; R&D Systems) and anti-actin antibody (1∶8,000; Sigma) were used for Western blot.

### Statistics

Statistical analysis was performed using Student's *t* test. A *p* value less than 0.05 was considered to be statistically significant. All results are represented as mean ± SEM.

## Supporting Information

Figure S1 Midbrain vasculature at 1.5 dpf. (A) Projected confocal image of a 1.5-dpf *Tg(kdrl:eGFP)* larva showing that some angiogenic sprouts (green arrows) were observed in the midbrain. Inset 1, filopodium-like sprout; Insets 2 and 3, sprouts with an expanded tip. Red and green arrows point to sprouts originated from the choroidal vascular plexus (CVP) or midbrain vasculature, respectively. The dashed square delineates the midbrain position. Scale, 50 µm. (B) 3-D reconstruction of the midbrain vasculature shown in (A). Yellow, basal communicating artery (BCA); white, midbrain vessels; blue, CVP.
(TIF)

Figure S2 Developmental expansion of midbrain vasculature during 2.0 to 7.5 dpf. Projected confocal images (left) and 3-D reconstruction (middle, right) of a larva's midbrain vasculature imaged at 2.0, 2.5, 3.0, 3.5, 4.5, and 7.5 dpf. The dashed square delineates the midbrain position. The segments that were pruned at the next time point are marked in red (middle). The newly formed segments after 2.0 dpf through angiogenesis are marked in green (right). Yellow, BCA; white, midbrain vasculature; blue, CVP. Scale, 50 µm.
(TIF)

Figure S3 Angiogenesis in the midbrain vasculature. Serial images show the process of vessel ingression from the CVP (yellow arrowheads) into the midbrain. The green arrows point to the tip of angiogenic sprouts. Scale, 10 µm.
(TIF)

Figure S4 Schematic of vessel segment Strahler order and internal loop. (A) Schematic of a complex vascular network with higher segment Strahler order and many internal vessel loops. The white arrows mark segments that are eliminated in (B). (B) Schematic showing reduction of segment Strahler order and loops that accompanies segment elimination. The numbers indicate the Strahler order of each segment. The 1^st^ Strahler order is assigned to segments that directly link with the CVP, and the orders of other vessel segments are defined following the Strahler ordering method. White dashed circles represent internal loops, which are defined as the nonoverlapping recurrent connections with a minimal number of segments.
(TIF)

Figure S5 Summary of changes in the percentage of segments located in the internal loop in the midbrain vasculature. The data were obtained from the same larvae analyzed in Figure 1D–G. * *p*<0.05; ** *p*<0.01; *** *p*<0.001 (paired Student's *t* test). Error bars, ± SEM.
(TIF)

Figure S6 Schematic of the blood flow direction in nearby unpruned segments. (A) For vessel pruning occurring in “H-type” vascular microcircuits, the two vessel segments at each end of pruned segments always exhibited the same blood flow direction (i, 87/87). “Not allowed” indicates that such situations do not exist in principle due to lack of blood flow output (iii) or input (vi). (B) For vessel pruning occurring in “O-type” vascular microcircuits, the pruned segment and its homology always showed same blood flow direction (i, 19/19). The dashed arrows indicate the direction of blood flow, and the red and blue lines represent pruned and unpruned vessel segments, respectively.
(TIF)

Figure S7 Calculation of blood cell velocity with kymograph. (A) An original Kymograph with two blood cells (1, 2). (B) Segmentation of the blood cells shown in (A) with a user-defined threshold and noise removal. (C) Automated velocity calculation based on the slope of the middle line (red) of segmented blood cells. The connected region of each segmented blood cell was first extracted. The coordinates of intersection points between each connected region and its image boundaries were then obtained. The average coordinates of intersection points at both the left and right boundaries for each connected region were linked (red) to calculate the velocity of corresponding blood cells. Scales, 8.99 µm (*x*-axis), 67.76 ms (*y*-axis).
(TIF)

Figure S8 Effects of norepinephrine bitartrate (NB) treatment-induced suppression of vessel pruning on global blood flow in the midbrain. Mean (A) and coefficient of variation (B) of blood flow velocity among midbrain vessel segments of control (Ctrl) or NB-treated zebrafish larvae at 4 dpf. NB (60 µM) was applied during 2–3.5 dpf to block vessel pruning (see Figure 5) and washed out at 3.5 dpf for heartbeat recovering. The flow velocity was measured at 4 dpf. At 4 dpf, the heartbeat of NB-treated larvae was no difference with that of control larvae (147±2 versus 151±2/min, *p*>0.05). The number on the bar represents the number of zebrafish larvae examined. For individual larvae, a mean value was averaged from more than 16 vessel segments. n.s., no significance; * *p*<0.05 (Student's *t* test). Error bars, ± SEM.
(TIF)

Figure S9 Verification of the measurement of blood cell velocity. (A) Projected images of the trunk vasculature in a double transgenic zebrafish *Tg(PU.1:gal4-uas-GFP,gata1:DsRed)* larva at 4 dpf. Dashed lines delineate the dorsal aorta (DA), posterior cardinal vein (PCV), and intersegmental vessel (ISV). Top, GFP signal; middle, DsRed signal; bottom, merged signal. Scale, 40 µm. (B) Kymographs of blood cells in 4-dpf midbrain vessels by measuring GFP (left) and DsRed (middle) signals. Right, merged. Blue lines mark blood cells expressing both GFP and DsRed. Scales, 5.43 µm (*x*-axis), 79 ms (*y*-axis). (C) Comparison of blood flow velocity measured with GFP- or DsRed-expressing blood cells in midbrain vessels. Each point represents the mean velocity of blood flow in one vessel segment, and the data from the same vessel are connected by a line. The mean velocity of each segment was averaged from 100±16 blood cells. The data were obtained from 15 segments in 2 larvae. n.s., no significance (paired Student's *t* test). Error bars, ± SEM.
(TIF)

Figure S10 Measurement of plasma flow velocity and its relationship with the occurrence of vessel pruning. (A) Serial images showing a vessel pruning event in the midbrain vasculature of a 2-dpf *Tg(kdrl:eGFP)* larva, which received microinjection of Fluosphere with green fluorescence (0.5 µm in diameter) into its circulation system. Fluosphere (green) and Dextran (red fluorescence, 10,000 MW) were co-injected into the circulation between –0:43 (hour:minute) and 0:00. The red and blue lines in the first panel indicate the site where axial line scanning was performed on a pruned (red arrow) and its adjacent unpruned segments, respectively. Scale, 20 µm. (B) Fluosphere-based kymographs showing bi-directional plasma flow in the pruned segment (right) and uni-directional flow in its adjacent unpruned segment (left). Scales, 5.43 µm (*x*-axis), 77.78 ms (*y*-axis). (C and D) Fluosphere-based calculation of plasma flow velocity (C) and shear stress (D) in pruned (red) and its adjacent unpruned segment (blue). The number on the bar represents the number of vessel segments examined. * *p*<0.05 (Student's *t* test). Error bars, ± SEM.
(TIF)

Figure S11 Effects of norepinephrine bitartrate treatment on heartbeat and midbrain blood flow. Effects of norepinephrine bitartrate (NB) treatment at 2 dpf for 24 h on the heartbeat (A; 139.3±2.8/min in control group, 166.3±2.1/min in NT-treated group) and the average velocity of midbrain blood flow (B; 0.26±0.02 mm/s in control group, 0.38±0.04 mm/s in NT-treated group) measured at 3 dpf. The numbers on the bars in (A) and (B) represent the numbers of larvae or vessel segments examined, respectively. The data in (B) were obtained from 15 larvae for control group and 16 larvae for NB treatment group. * *p*<0.05; *** *p*<0.001 (Student's *t* test). Error bars, ± SEM.
(TIF)

Figure S12 Norepinephrine bitartrate treatment increases both the segment Strahler order and internal loop number. (A and B) Representative centerlines of 3 d post-fertilization midbrain vasculature under control (A) and norepinephrine bitartrate treatment (B, NB). Red, orange, yellow, green, and cyan mark vessel segments with the 1^st^–5^th^ Strahler order, respectively. The white lines indicate internal vessel loops, and the white dots represent branch points between the CVP and midbrain vessel segments. (C and D) Summary of data showing that NB treatment increases both segment Strahler order (C) and internal loop number (D) of the midbrain vasculature. * *p*<0.05 (Student's *t* test). Error bars, ± SEM.
(TIF)

Figure S13 Effects of heartbeat suppression on vessel pruning. (A–C) Projected images of zebrafish larval midbrain vasculature at 50 hpf. Larvae were treated with normal solution (Ctrl, A), MS222 (tricaine, 0.66 mg/ml; B), or 2,3-butanedione-2-monoxime (20 mM, BDM; C) from 48 hpf and imaged at 50 hpf. (D and E) Projected images of zebrafish larval midbrain vasculature at 2 dpf. Larvae were microinjected with 4 ng control morpholino (Ctrl MO; D) or 4 ng *Tnnt2* MO (E). Scale, 50 µm. (F–G) Time-lapse serial imaging showing BDM-induced vessel pruning. The morphology of vessels in the whole midbrain (F) and highlighted area (G) were shown before BDM treatment and after the onset of BDM treatment. BDM was bath-applied during 2–2.5 dpf and imaging was performed during this period. The regressed segments are pointed by the red arrows in the real images or marked in red in the 3-D reconstruction (G). Time, hour:minute. Scales, 50 µm in (F) and 20 µm in (G).
(TIF)

Figure S14 TUNEL staining of developing zebrafish midbrain. (A) TUNEL staining of DNase I-treated *Tg(kdrl:eGFP)* zebrafish brain at 3 dpf. DNase I treatment generates strand breaks in the DNA to provide a positive TUNEL reaction. Red, TUNEL signal. The dashed white line delineates the outline of midbrain. (B) Staining of *Tg(kdrl:eGFP)* zebrafish larva brain without terminal transferase, serving as a negative control. (C and D) Two examples of TUNEL staining of *Tg(kdrl:eGFP)* zebrafish brain (WT1, WT2) at 3 dpf. Scale, 50 µm.
(TIF)

Figure S15 Effects of down-regulation of *VEGFA* and *Angiopoietin-2* on vessel pruning. (A) Western blotting showing that *VEGFA* MO reduces *VEGFA* expression. (B) RT-PCR analysis showing that *Angiopoietin-2 (Ang2)* splicing MO induces a shift from wild-type (blue asterisk) to mis-spliced transcripts of *Ang2* (red asterisk). (C–E) Projected midbrain vasculature images of 3-dpf *Tg(kdrl:eGFP)* zebrafish larvae injected with control MO (8 ng; C), *VEGFA* MO (2 ng; D), and *Ang2* MO (1 ng; E). (F) Summary of pruning ratio of midbrain vessel segments. The number on the bar in (F) represents the number of zebrafish larvae examined. Scale, 50 µm in (C–E). n.s., no significance; *** *p*<0.001 (Student's *t* test). Error bars, ± SEM.
(TIF)

Figure S16 Effects of DMOG treatment on vessel pruning. (A) *o*-Dianisidine staining showing that DMOG treatment increases the amount of blood cells in treated embryos. DMSO (0.2%) or DMOG (0.2 mM) was bath-applied during 2–3 dpf. (B and C) Effect of DMOG treatment on vessel pruning of larval zebrafish midbrain. Projected confocal images showing midbrain vasculature of DMSO- (B) and DMOG-treated (C) zebrafish larvae at 3 dpf. (D) Average number of vessel pruning events occurring between 2 and 3 dpf in single larval zebrafish midbrain. Each small square in (D) represents the data obtained from single larvae. Scale, 50 µm. n.s., no significance (Student's *t* test). Error bars, ± SEM.
(TIF)

Figure S17 Method for tracing fate of each vessel segment in the midbrain. (A and B) Projected confocal images (left) and 3-D reconstructions (right) of half midbrain vasculature in a *Tg(kdrl:eGFP)* zebrafish larvae at 2.5 dpf (A) or 3 dpf (B). Colored balls mark different branch points. The dashed circle marks the site at which a branch point will appear at the next imaging time point. The corresponding movies of the 3-D rotation centerlines are shown in Video S7. Green, newly formed segments; red, pruning segments; blue, CVP. Scale, 50 µm.
(TIF)

Figure S18 Numerical simulation with constant and fluctuating flow. (A) Diagram of constant (black) and sinewave-like fluctuating blood flow (gray line) with the same mean value. (B) Simulated changes in the pressure drop (top) and radius (bottom) of predicted pruned vessel segments (color lines) when the constant (left) or fluctuating flow (right) is inputted into the mathematical model. The data obtained from each segment are marked with a distinct color. (C) Comparison of time steps required for the completion of vessel pruning. The data obtained from the same vessel segments are connected by a line. n.s., no significance (paired Student's *t* test).
(TIF)

Video S1 A *z*-stack movie obtained by a confocal microscope showing both the midbrain vasculature (green) and neural tissue (red) of a 3-dpf *Tg(kdrl:eGFP,HuC:gal4-uas-mCherry)* zebrafish larva. At the beginning of the movie, its projected image, which is shown in the bottom panel of Figure 1A, is presented. The direction of the movie is from ventral to dorsal. Dorsal view, anterior is down.
(MOV)

Video S2 3-D rotation of a 2-dpf midbrain vasculature centerline. At the beginning of the movie, its projected image, which is shown in the top panel of Figure 1C, is presented. The clockwise rotation begins from a dorsal view with caudal up and stops after one cycle.
(MOV)

Video S3 3-D rotation of a 4-dpf midbrain vasculature centerline. At the beginning of the movie, its projected image, which is shown in the middle panel of Figure 1C, is presented. The clockwise rotation begins from a dorsal view with caudal up and stops after one cycle.
(MOV)

Video S4 3-D rotation of a 7.5-dpf midbrain vasculature centerline. At the beginning of the movie, its projected image, which is shown in the bottom panel of Figure 1C, is presented. The clockwise rotation begins from a dorsal view with caudal up and stops after one cycle.
(MOV)

Video S5 A time-lapse movie obtained by a confocal microscope showing blood flow in a pruned and an adjacent unpruned vessel segment. A corresponding image is shown in the first panel of Figure 5A (dashed square). In the movie, the vessel segment in the top region will undergo pruning, whereas the one in the left region will not. The yellow arrowhead marks a blood cell with bi-directional movement in the segment that will be pruned. Images were captured every 110 ms and presented at 3 frames per second (fps). The real total duration of the movie is 23.1 s. The contrast and brightness of the video were adjusted for clarity.
(MOV)

Video S6 A time-lapse movie obtained by a confocal microscope showing that the blood flow of a vessel segment is not completely occluded by a bead. The region of time-lapse scanning is indicated in the first panel of Figure 5H (dash square). The images were captured every 124 ms and presented at 4 fps. The real total duration of the movie is 31.8 s. The contrast and brightness of the video were adjusted for clarity.
(MOV)

Video S7 3-D rotation of the centerlines of a half midbrain vasculature imaged at 2.5 and 3.0 dpf. At the beginning of the movie, projected images, which are shown in the right panels of Figure S17A and S17B, are presented. The clockwise rotation begins from a dorsal view with caudal up and stops after one cycle.
(MOV)

## Abbreviations

3-D: three-dimensional
BBB: blood-brain barrier
BCA: basal communicating artery
CV: coefficient of variation
CVP: choroidal vascular plexus
dpf: days post-fertilization
EC: endothelial cell
GFP: green fluorescent protein
MO: morpholino oligonucleotide
NB: norepinephrine bitartrate
TUNEL: terminal deoxynucleotidyl transferase dUTP nick end labeling
