# Supplementary figures and images for: Haemodynamics-Driven Developmental Pruning of Brain Vasculature in Zebrafish
Source: PLoS Biol. 2012 Aug 14;10(8):e1001374. doi: 10.1371/journal.pbio.1001374 (PMC3419171; doi:10.1371/journal.pbio.1001374)

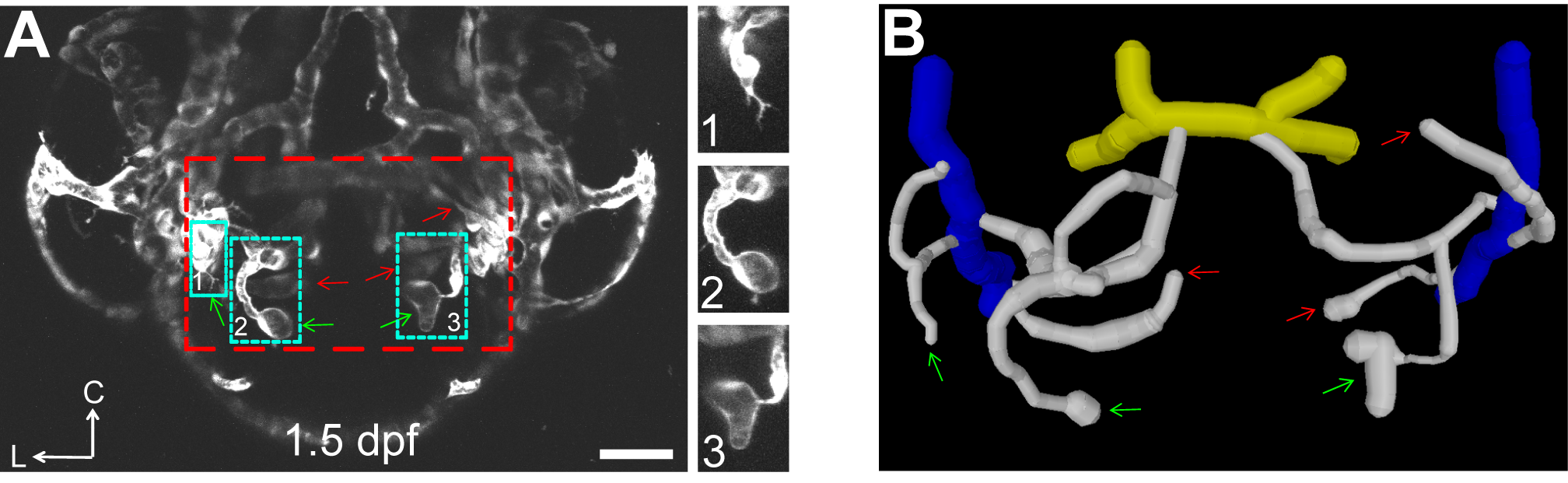

Supplement: Figure S1 — Midbrain vasculature at 1.5 dpf. (A) Projected confocal image of a 1.5-dpf Tg(kdrl:eGFP) larva showing that some angiogenic sprouts (green arrows) were observed in the midbrain. Inset 1, filopodium-like sprout; Insets 2 and 3, sprouts with an expanded tip. Red and green arrows point to sprouts originated from the choroidal vascular plexus (CVP) or midbrain vasculature, respectively. The dashed square delineates the midbrain position. Scale, 50 µm. (B) 3-D reconstruction of the midbrain vasculature shown in (A). Yellow, basal communicating artery (BCA); white, midbrain vessels; blue, CVP. (TIF) [file pbio.1001374.s001.tif]

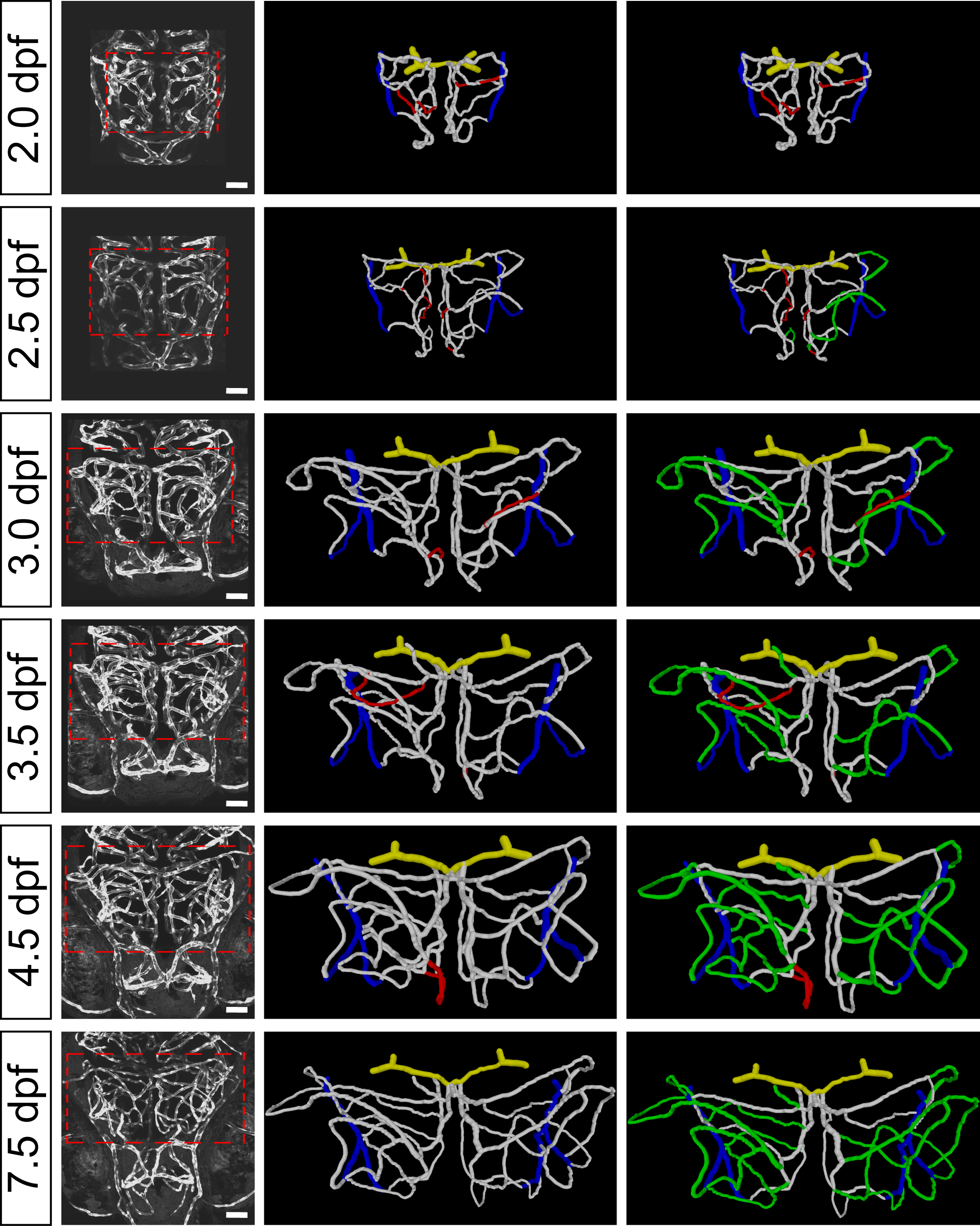

Supplement: Figure S2 — Developmental expansion of midbrain vasculature during 2.0 to 7.5 dpf. Projected confocal images (left) and 3-D reconstruction (middle, right) of a larva's midbrain vasculature imaged at 2.0, 2.5, 3.0, 3.5, 4.5, and 7.5 dpf. The dashed square delineates the midbrain position. The segments that were pruned at the next time point are marked in red (middle). The newly formed segments after 2.0 dpf through angiogenesis are marked in green (right). Yellow, BCA; white, midbrain vasculature; blue, CVP. Scale, 50 µm. (TIF) [file pbio.1001374.s002.tif]

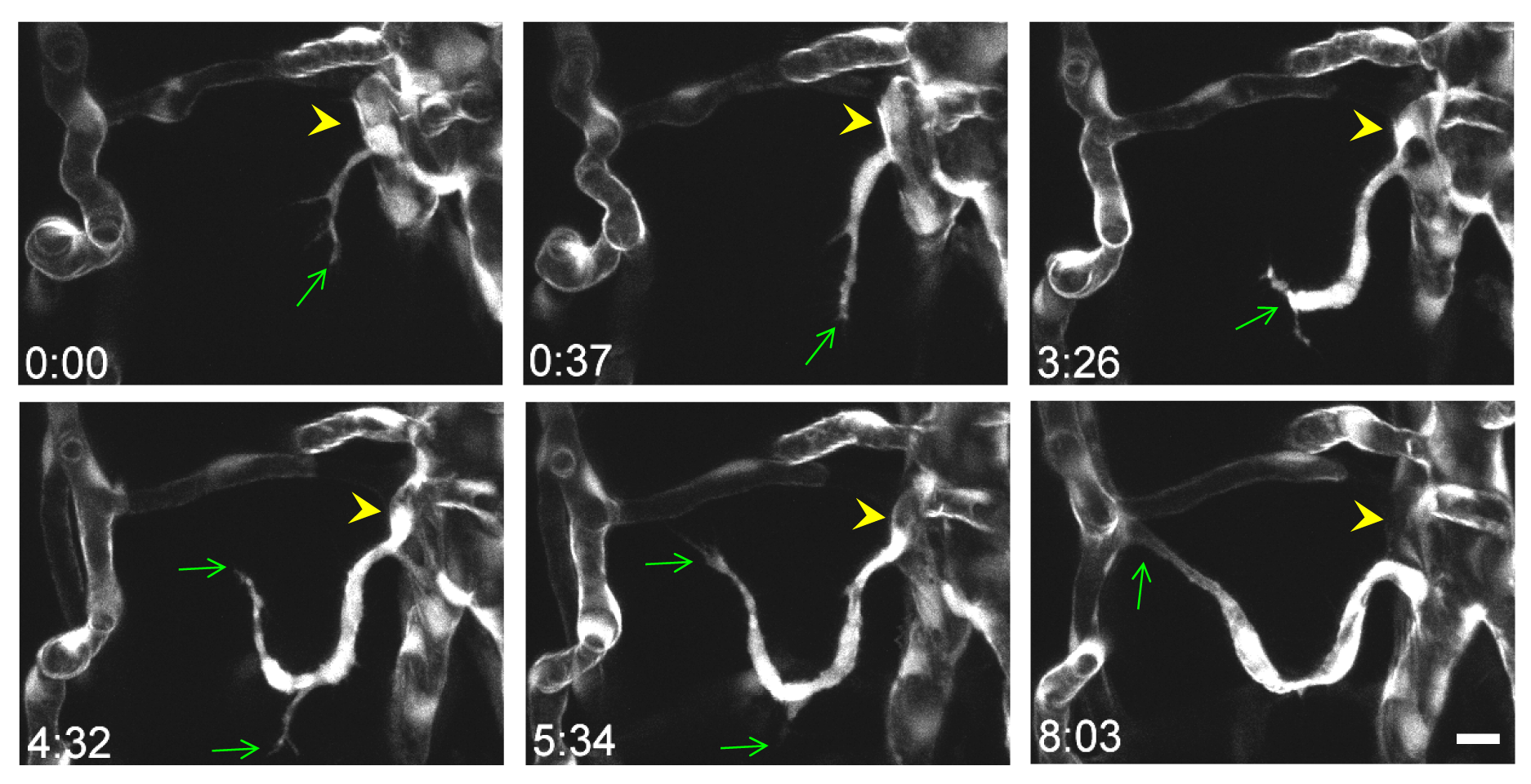

Supplement: Figure S3 — Angiogenesis in the midbrain vasculature. Serial images show the process of vessel ingression from the CVP (yellow arrowheads) into the midbrain. The green arrows point to the tip of angiogenic sprouts. Scale, 10 µm. (TIF) [file pbio.1001374.s003.tif]

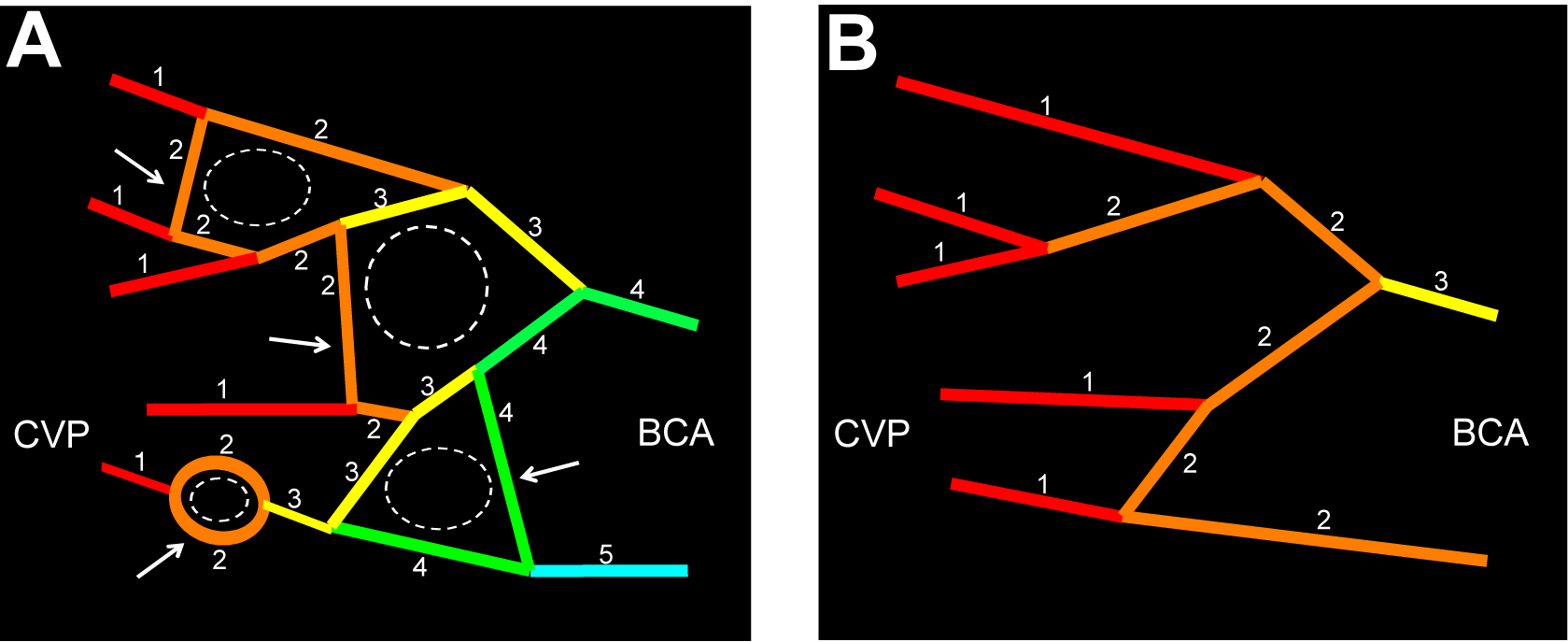

Supplement: Figure S4 — Schematic of vessel segment Strahler order and internal loop. (A) Schematic of a complex vascular network with higher segment Strahler order and many internal vessel loops. The white arrows mark segments that are eliminated in (B). (B) Schematic showing reduction of segment Strahler order and loops that accompanies segment elimination. The numbers indicate the Strahler order of each segment. The 1st Strahler order is assigned to segments that directly link with the CVP, and the orders of other vessel segments are defined following the Strahler ordering method. White dashed circles represent internal loops, which are defined as the nonoverlapping recurrent connections with a minimal number of segments. (TIF) [file pbio.1001374.s004.tif]

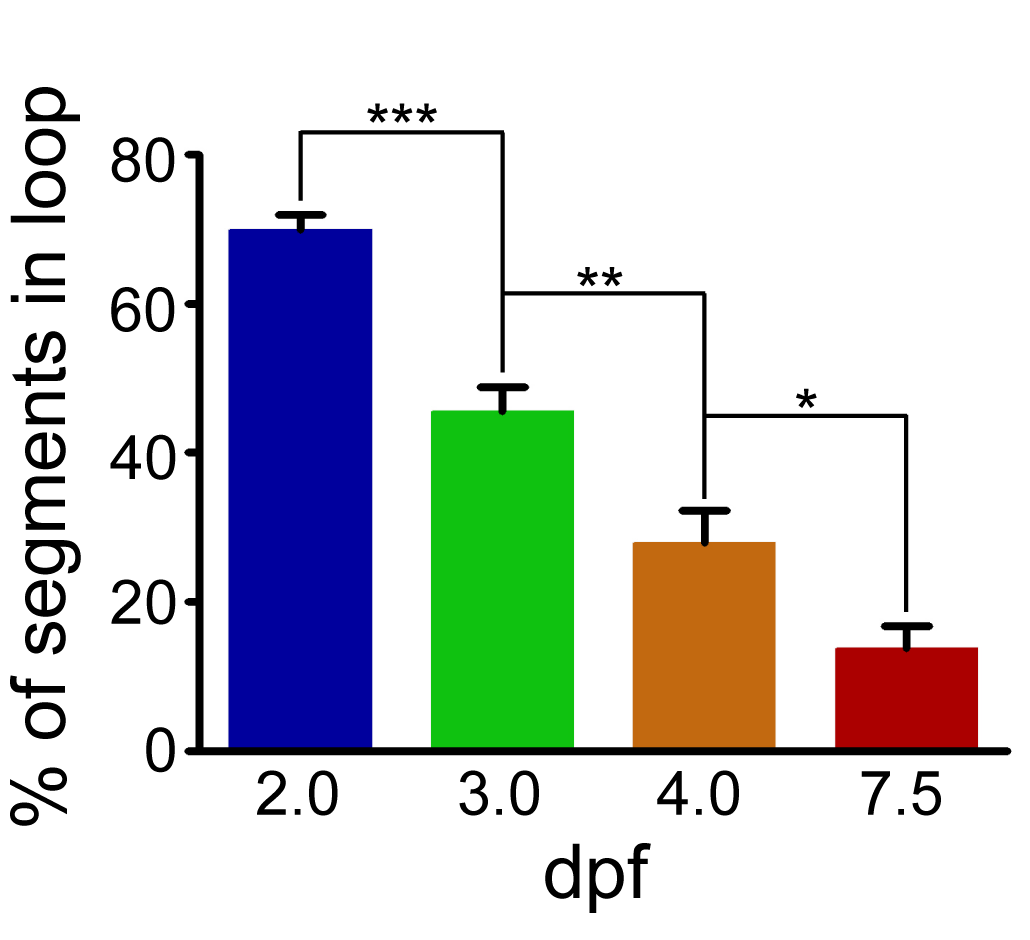

Supplement: Figure S5 — Summary of changes in the percentage of segments located in the internal loop in the midbrain vasculature. The data were obtained from the same larvae analyzed in Figure 1D–G. * p<0.05; ** p<0.01; *** p<0.001 (paired Student's t test). Error bars, ± SEM. (TIF) [file pbio.1001374.s005.tif]

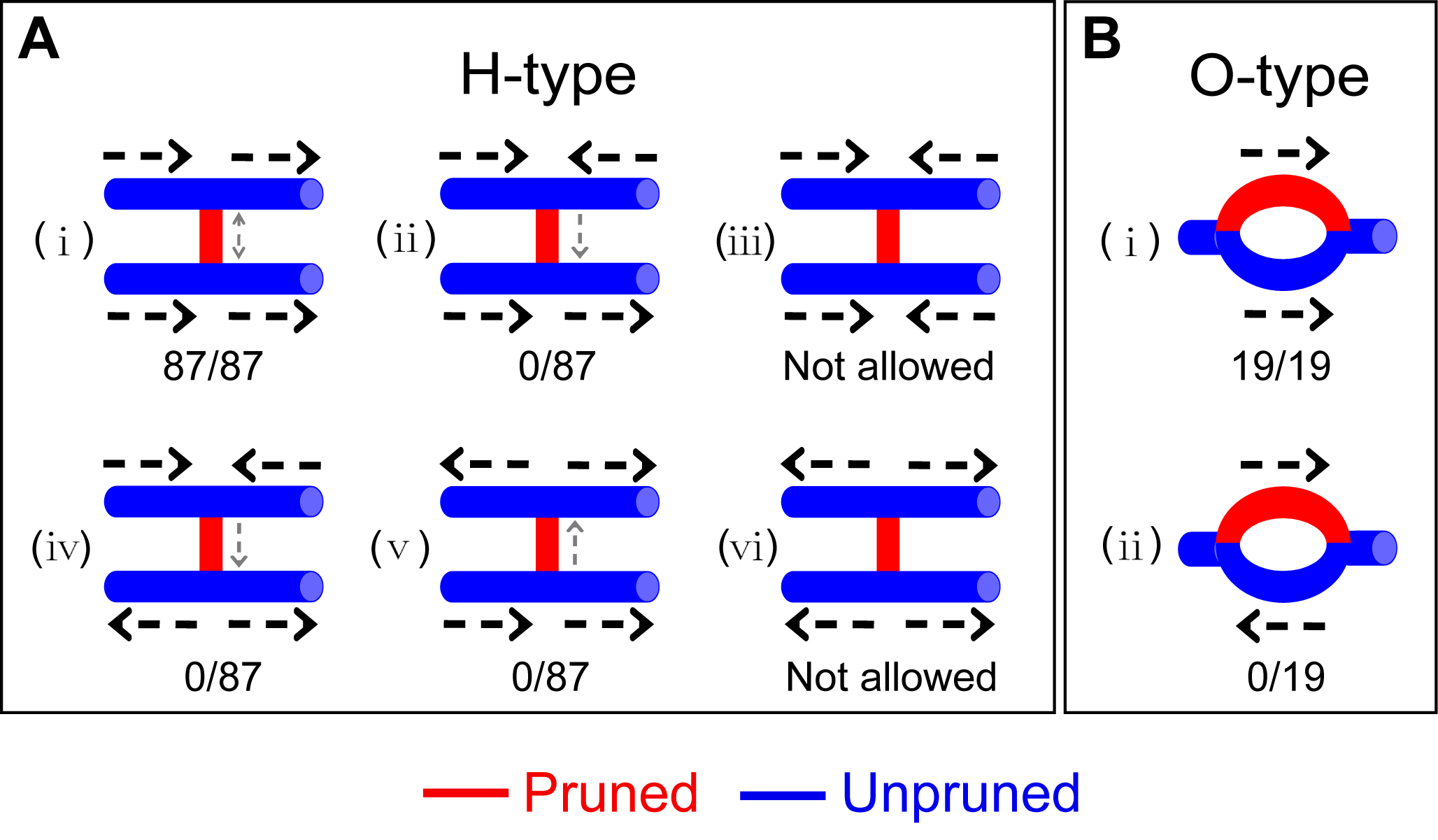

Supplement: Figure S6 — Schematic of the blood flow direction in nearby unpruned segments. (A) For vessel pruning occurring in “H-type” vascular microcircuits, the two vessel segments at each end of pruned segments always exhibited the same blood flow direction (i, 87/87). “Not allowed” indicates that such situations do not exist in principle due to lack of blood flow output (iii) or input (vi). (B) For vessel pruning occurring in “O-type” vascular microcircuits, the pruned segment and its homology always showed same blood flow direction (i, 19/19). The dashed arrows indicate the direction of blood flow, and the red and blue lines represent pruned and unpruned vessel segments, respectively. (TIF) [file pbio.1001374.s006.tif]

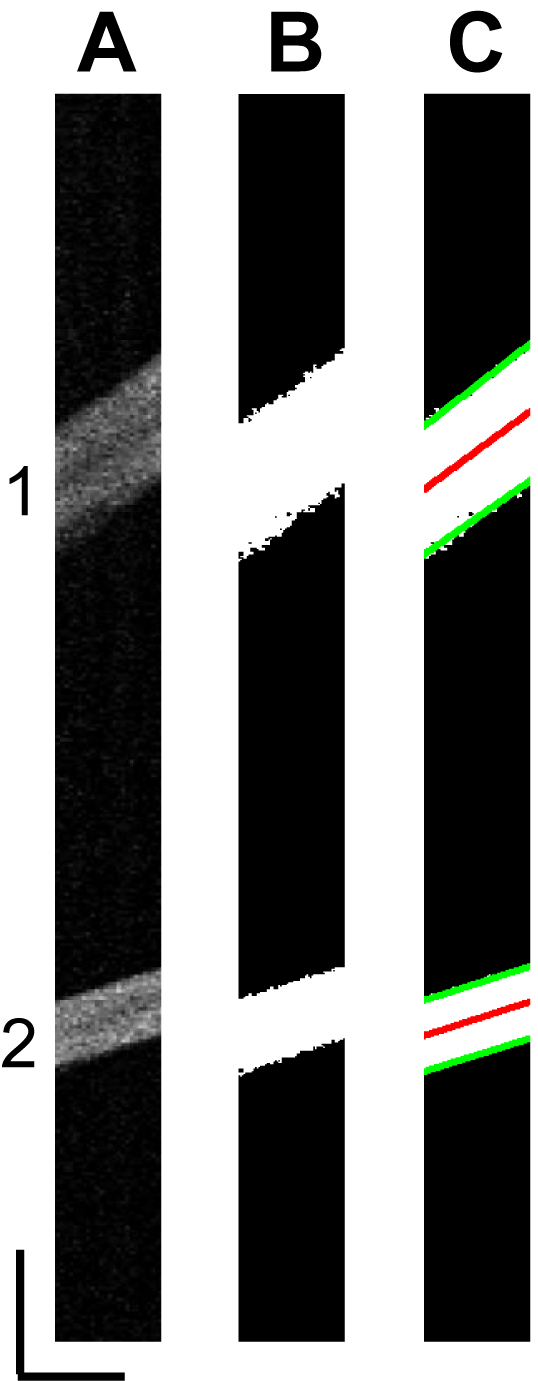

Supplement: Figure S7 — Calculation of blood cell velocity with kymograph. (A) An original Kymograph with two blood cells (1, 2). (B) Segmentation of the blood cells shown in (A) with a user-defined threshold and noise removal. (C) Automated velocity calculation based on the slope of the middle line (red) of segmented blood cells. The connected region of each segmented blood cell was first extracted. The coordinates of intersection points between each connected region and its image boundaries were then obtained. The average coordinates of intersection points at both the left and right boundaries for each connected region were linked (red) to calculate the velocity of corresponding blood cells. Scales, 8.99 µm (x-axis), 67.76 ms (y-axis). (TIF) [file pbio.1001374.s007.tif]

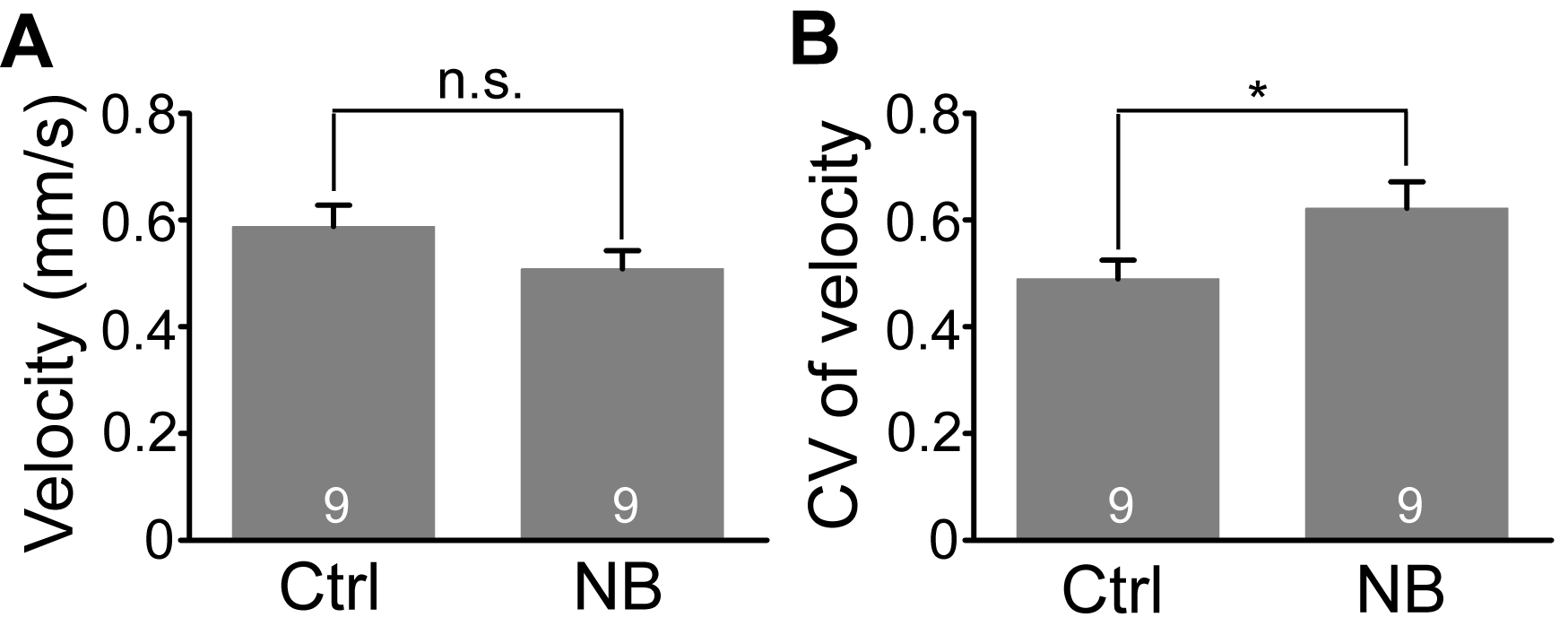

Supplement: Figure S8 — Effects of norepinephrine bitartrate (NB) treatment-induced suppression of vessel pruning on global blood flow in the midbrain. Mean (A) and coefficient of variation (B) of blood flow velocity among midbrain vessel segments of control (Ctrl) or NB-treated zebrafish larvae at 4 dpf. NB (60 µM) was applied during 2–3.5 dpf to block vessel pruning (see Figure 5) and washed out at 3.5 dpf for heartbeat recovering. The flow velocity was measured at 4 dpf. At 4 dpf, the heartbeat of NB-treated larvae was no difference with that of control larvae (147±2 versus 151±2/min, p>0.05). The number on the bar represents the number of zebrafish larvae examined. For individual larvae, a mean value was averaged from more than 16 vessel segments. n.s., no significance; * p<0.05 (Student's t test). Error bars, ± SEM. (TIF) [file pbio.1001374.s008.tif]

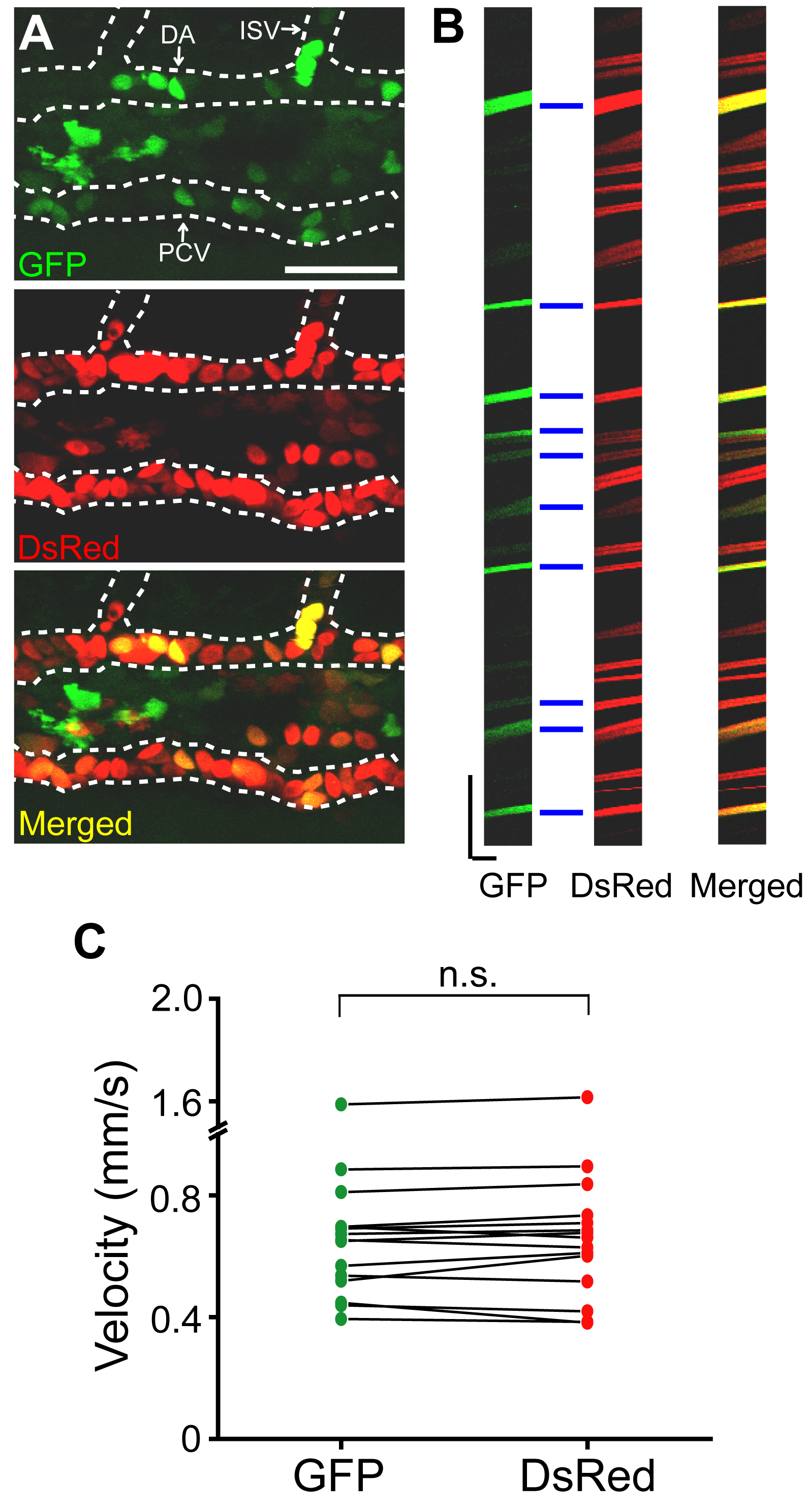

Supplement: Figure S9 — Verification of the measurement of blood cell velocity. (A) Projected images of the trunk vasculature in a double transgenic zebrafish Tg(PU.1:gal4-uas-GFP,gata1:DsRed) larva at 4 dpf. Dashed lines delineate the dorsal aorta (DA), posterior cardinal vein (PCV), and intersegmental vessel (ISV). Top, GFP signal; middle, DsRed signal; bottom, merged signal. Scale, 40 µm. (B) Kymographs of blood cells in 4-dpf midbrain vessels by measuring GFP (left) and DsRed (middle) signals. Right, merged. Blue lines mark blood cells expressing both GFP and DsRed. Scales, 5.43 µm (x-axis), 79 ms (y-axis). (C) Comparison of blood flow velocity measured with GFP- or DsRed-expressing blood cells in midbrain vessels. Each point represents the mean velocity of blood flow in one vessel segment, and the data from the same vessel are connected by a line. The mean velocity of each segment was averaged from 100±16 blood cells. The data were obtained from 15 segments in 2 larvae. n.s., no significance (paired Student's t test). Error bars, ± SEM. (TIF) [file pbio.1001374.s009.tif]

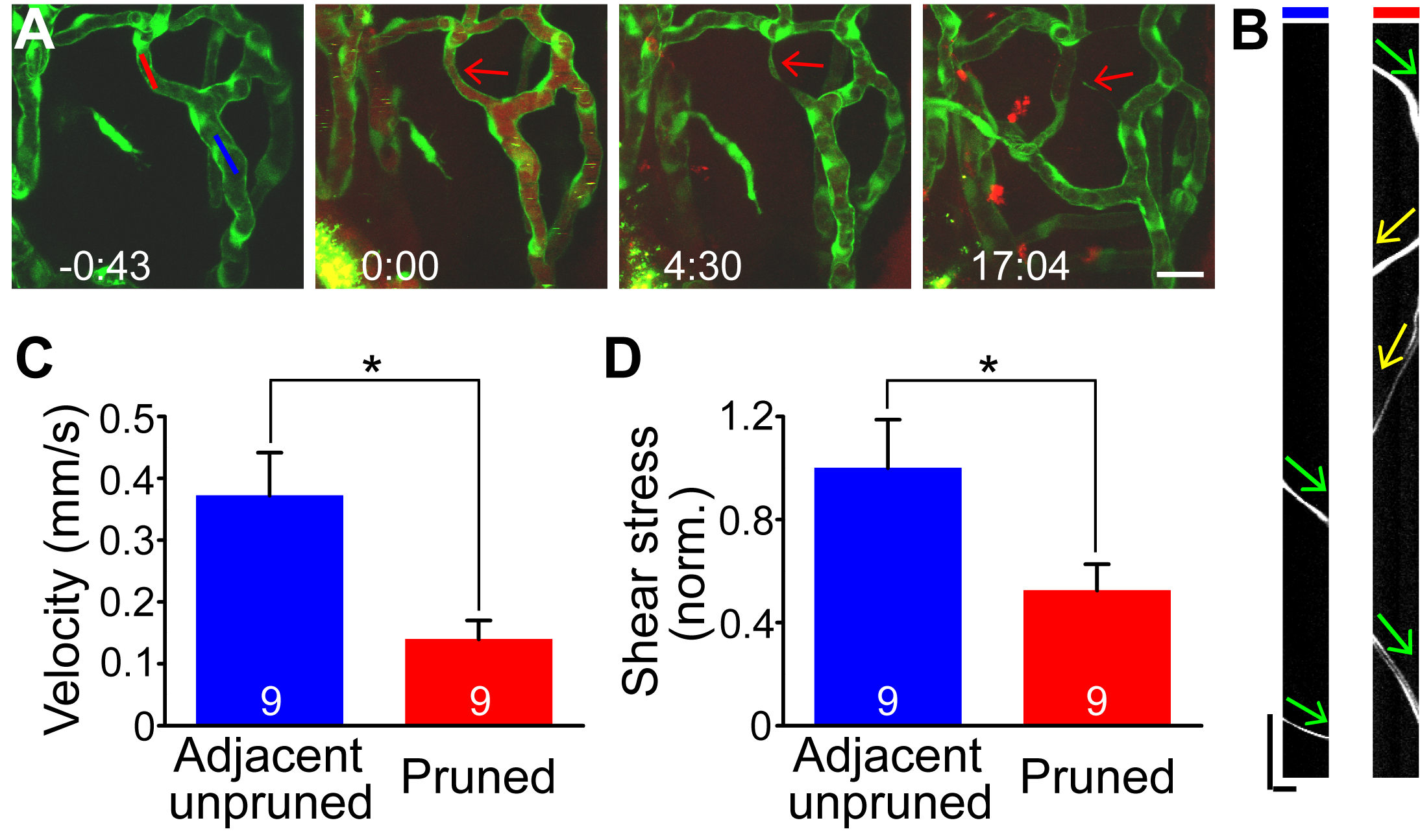

Supplement: Figure S10 — Measurement of plasma flow velocity and its relationship with the occurrence of vessel pruning. (A) Serial images showing a vessel pruning event in the midbrain vasculature of a 2-dpf Tg(kdrl:eGFP) larva, which received microinjection of Fluosphere with green fluorescence (0.5 µm in diameter) into its circulation system. Fluosphere (green) and Dextran (red fluorescence, 10,000 MW) were co-injected into the circulation between –0:43 (hour:minute) and 0:00. The red and blue lines in the first panel indicate the site where axial line scanning was performed on a pruned (red arrow) and its adjacent unpruned segments, respectively. Scale, 20 µm. (B) Fluosphere-based kymographs showing bi-directional plasma flow in the pruned segment (right) and uni-directional flow in its adjacent unpruned segment (left). Scales, 5.43 µm (x-axis), 77.78 ms (y-axis). (C and D) Fluosphere-based calculation of plasma flow velocity (C) and shear stress (D) in pruned (red) and its adjacent unpruned segment (blue). The number on the bar represents the number of vessel segments examined. * p<0.05 (Student's t test). Error bars, ± SEM. (TIF) [file pbio.1001374.s010.tif]

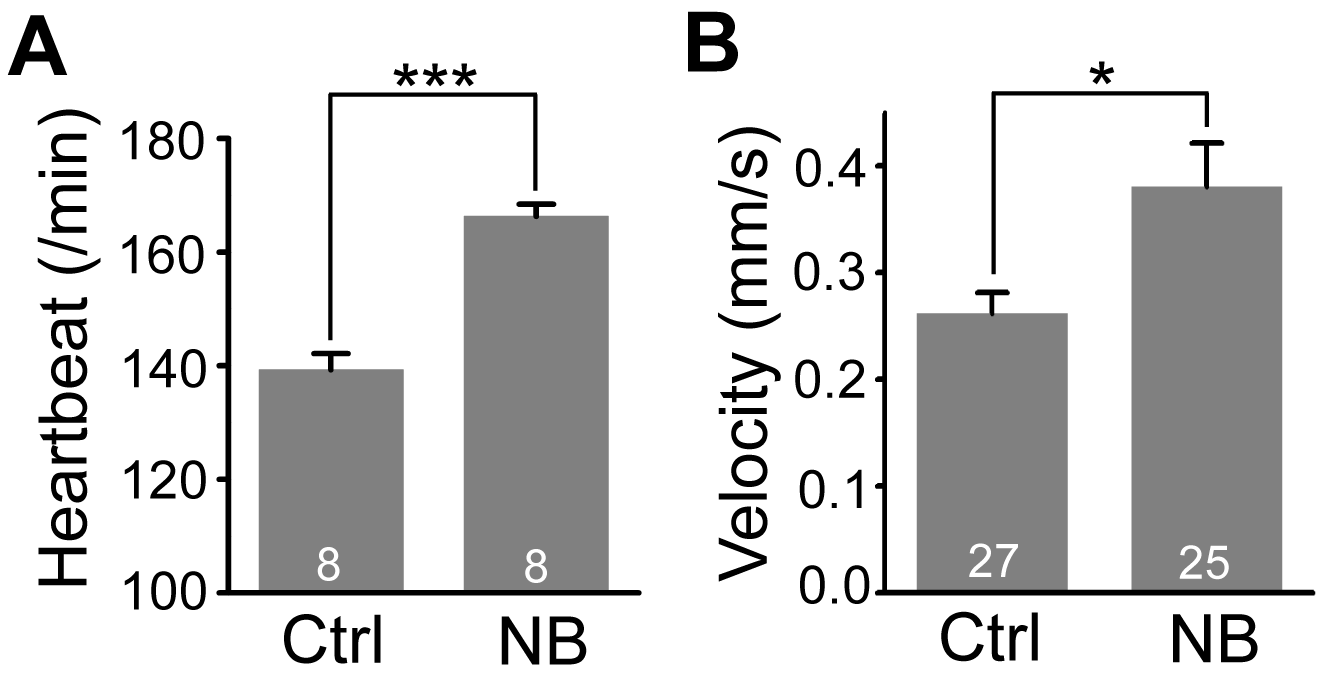

Supplement: Figure S11 — Effects of norepinephrine bitartrate treatment on heartbeat and midbrain blood flow. Effects of norepinephrine bitartrate (NB) treatment at 2 dpf for 24 h on the heartbeat (A; 139.3±2.8/min in control group, 166.3±2.1/min in NT-treated group) and the average velocity of midbrain blood flow (B; 0.26±0.02 mm/s in control group, 0.38±0.04 mm/s in NT-treated group) measured at 3 dpf. The numbers on the bars in (A) and (B) represent the numbers of larvae or vessel segments examined, respectively. The data in (B) were obtained from 15 larvae for control group and 16 larvae for NB treatment group. * p<0.05; *** p<0.001 (Student's t test). Error bars, ± SEM. (TIF) [file pbio.1001374.s011.tif]

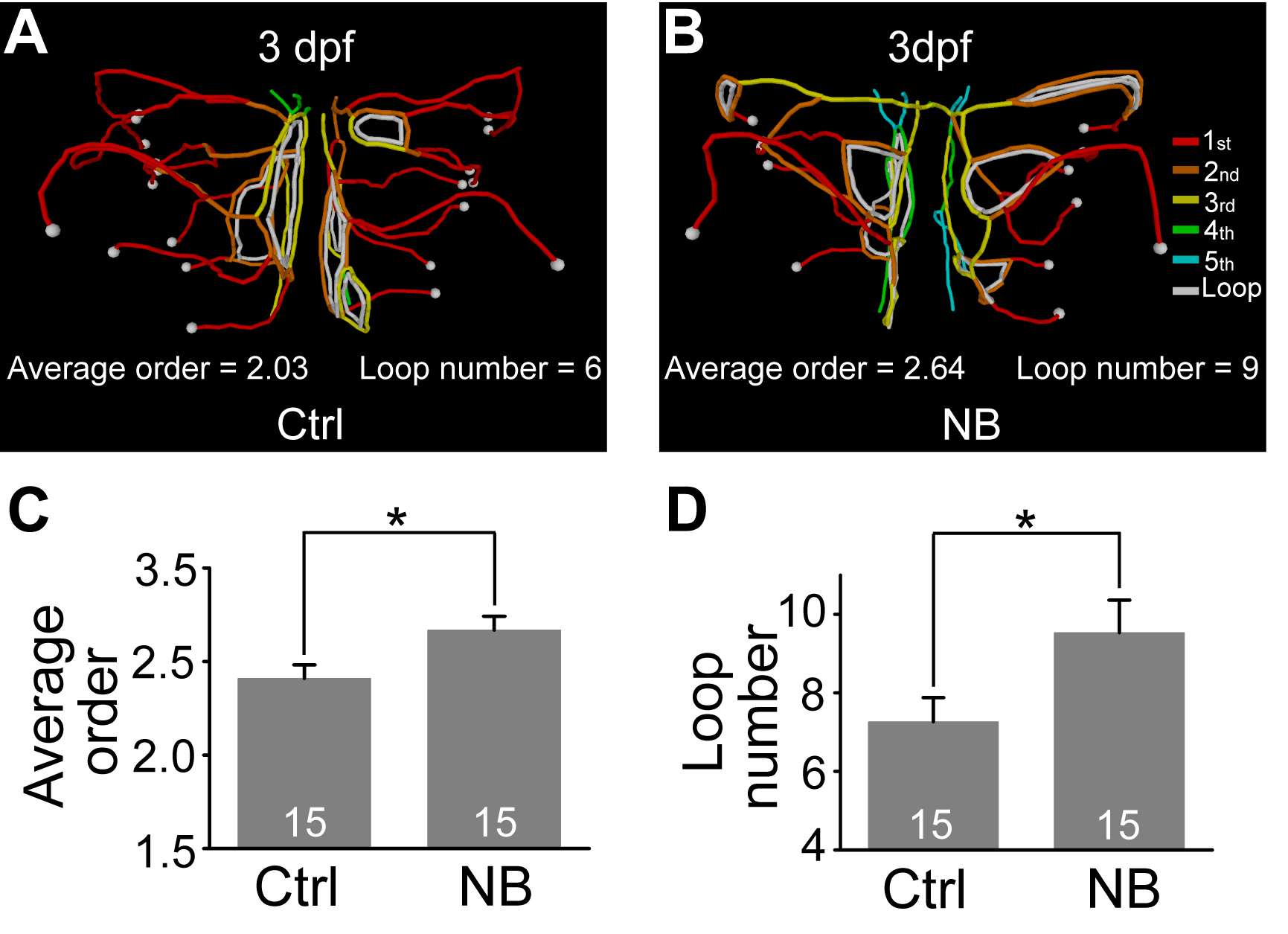

Supplement: Figure S12 — Norepinephrine bitartrate treatment increases both the segment Strahler order and internal loop number. (A and B) Representative centerlines of 3 d post-fertilization midbrain vasculature under control (A) and norepinephrine bitartrate treatment (B, NB). Red, orange, yellow, green, and cyan mark vessel segments with the 1st–5th Strahler order, respectively. The white lines indicate internal vessel loops, and the white dots represent branch points between the CVP and midbrain vessel segments. (C and D) Summary of data showing that NB treatment increases both segment Strahler order (C) and internal loop number (D) of the midbrain vasculature. * p<0.05 (Student's t test). Error bars, ± SEM. (TIF) [file pbio.1001374.s012.tif]

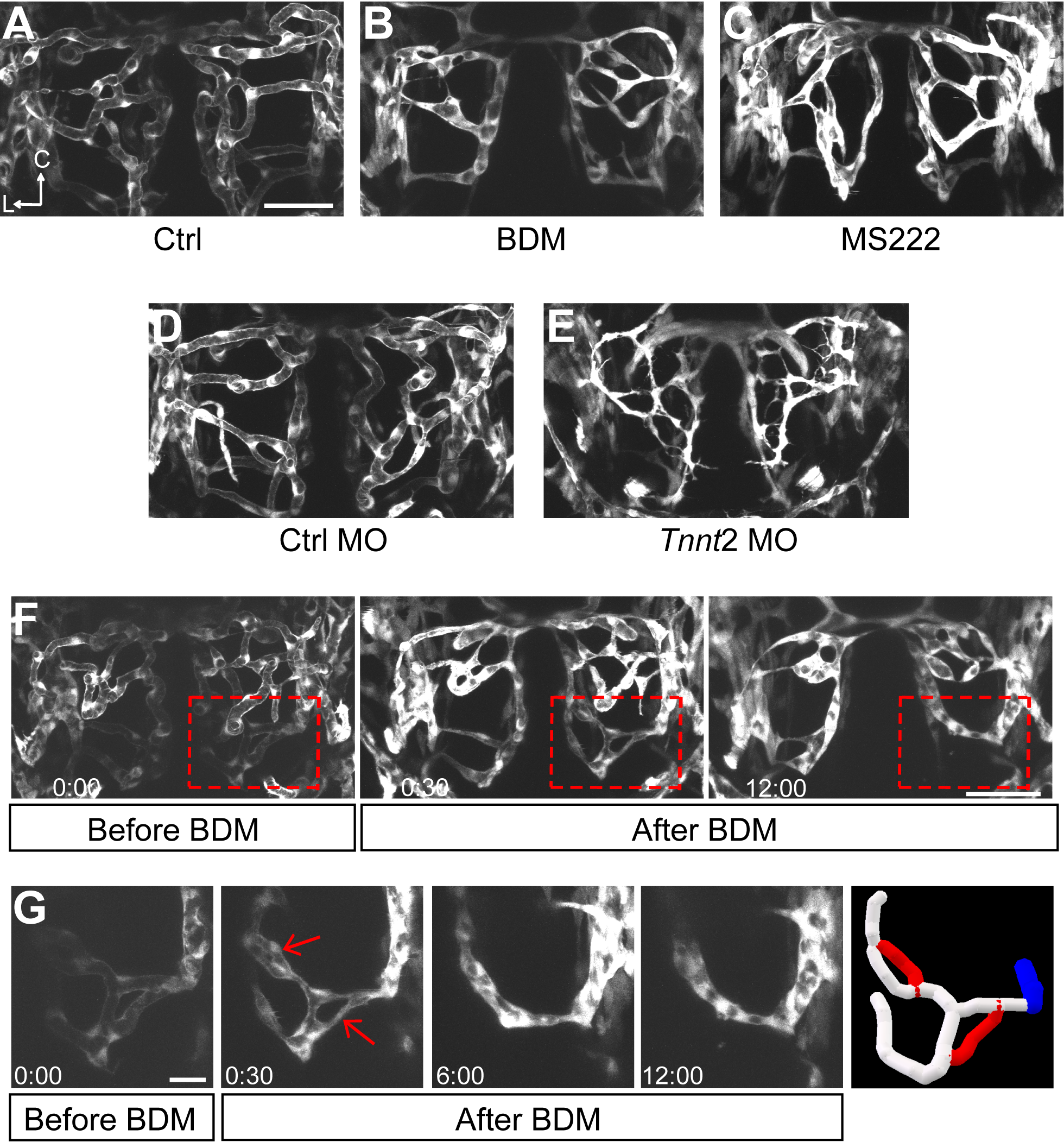

Supplement: Figure S13 — Effects of heartbeat suppression on vessel pruning. (A–C) Projected images of zebrafish larval midbrain vasculature at 50 hpf. Larvae were treated with normal solution (Ctrl, A), MS222 (tricaine, 0.66 mg/ml; B), or 2,3-butanedione-2-monoxime (20 mM, BDM; C) from 48 hpf and imaged at 50 hpf. (D and E) Projected images of zebrafish larval midbrain vasculature at 2 dpf. Larvae were microinjected with 4 ng control morpholino (Ctrl MO; D) or 4 ng Tnnt2 MO (E). Scale, 50 µm. (F–G) Time-lapse serial imaging showing BDM-induced vessel pruning. The morphology of vessels in the whole midbrain (F) and highlighted area (G) were shown before BDM treatment and after the onset of BDM treatment. BDM was bath-applied during 2–2.5 dpf and imaging was performed during this period. The regressed segments are pointed by the red arrows in the real images or marked in red in the 3-D reconstruction (G). Time, hour:minute. Scales, 50 µm in (F) and 20 µm in (G). (TIF) [file pbio.1001374.s013.tif]

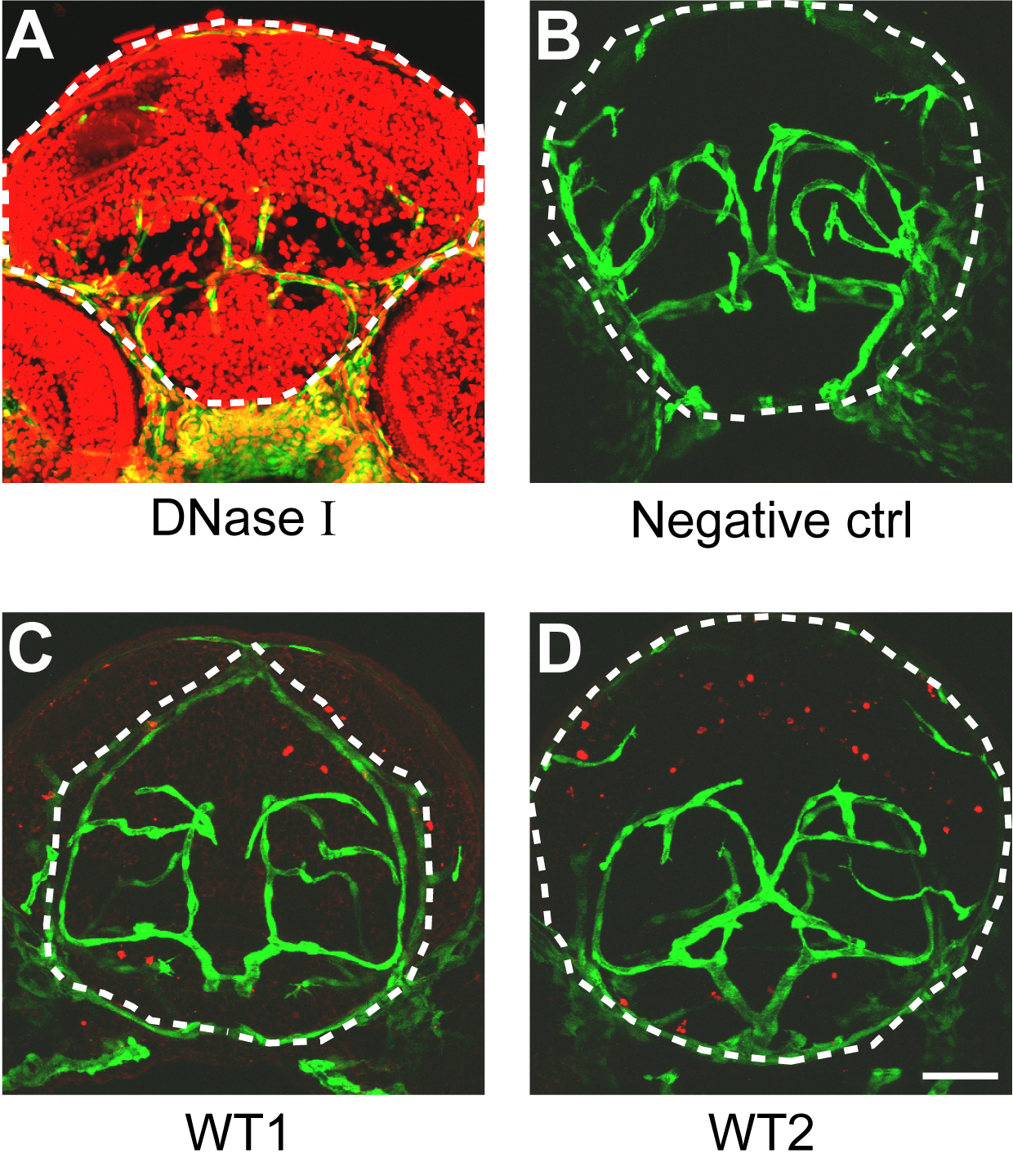

Supplement: Figure S14 — TUNEL staining of developing zebrafish midbrain. (A) TUNEL staining of DNase I-treated Tg(kdrl:eGFP) zebrafish brain at 3 dpf. DNase I treatment generates strand breaks in the DNA to provide a positive TUNEL reaction. Red, TUNEL signal. The dashed white line delineates the outline of midbrain. (B) Staining of Tg(kdrl:eGFP) zebrafish larva brain without terminal transferase, serving as a negative control. (C and D) Two examples of TUNEL staining of Tg(kdrl:eGFP) zebrafish brain (WT1, WT2) at 3 dpf. Scale, 50 µm. (TIF) [file pbio.1001374.s014.tif]

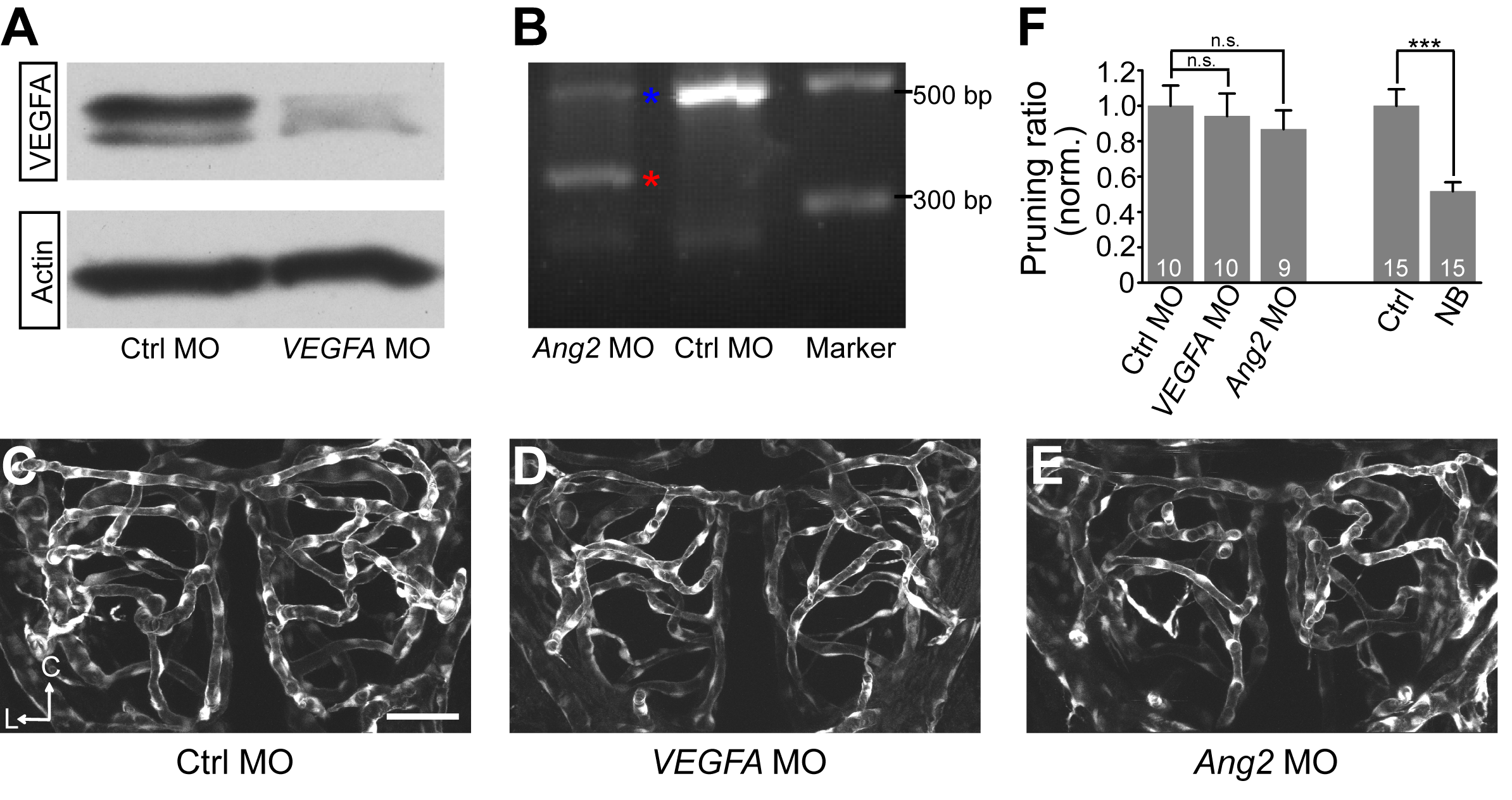

Supplement: Figure S15 — Effects of down-regulation of VEGFA and Angiopoietin-2 on vessel pruning. (A) Western blotting showing that VEGFA MO reduces VEGFA expression. (B) RT-PCR analysis showing that Angiopoietin-2 (Ang2) splicing MO induces a shift from wild-type (blue asterisk) to mis-spliced transcripts of Ang2 (red asterisk). (C–E) Projected midbrain vasculature images of 3-dpf Tg(kdrl:eGFP) zebrafish larvae injected with control MO (8 ng; C), VEGFA MO (2 ng; D), and Ang2 MO (1 ng; E). (F) Summary of pruning ratio of midbrain vessel segments. The number on the bar in (F) represents the number of zebrafish larvae examined. Scale, 50 µm in (C–E). n.s., no significance; *** p<0.001 (Student's t test). Error bars, ± SEM. (TIF) [file pbio.1001374.s015.tif]

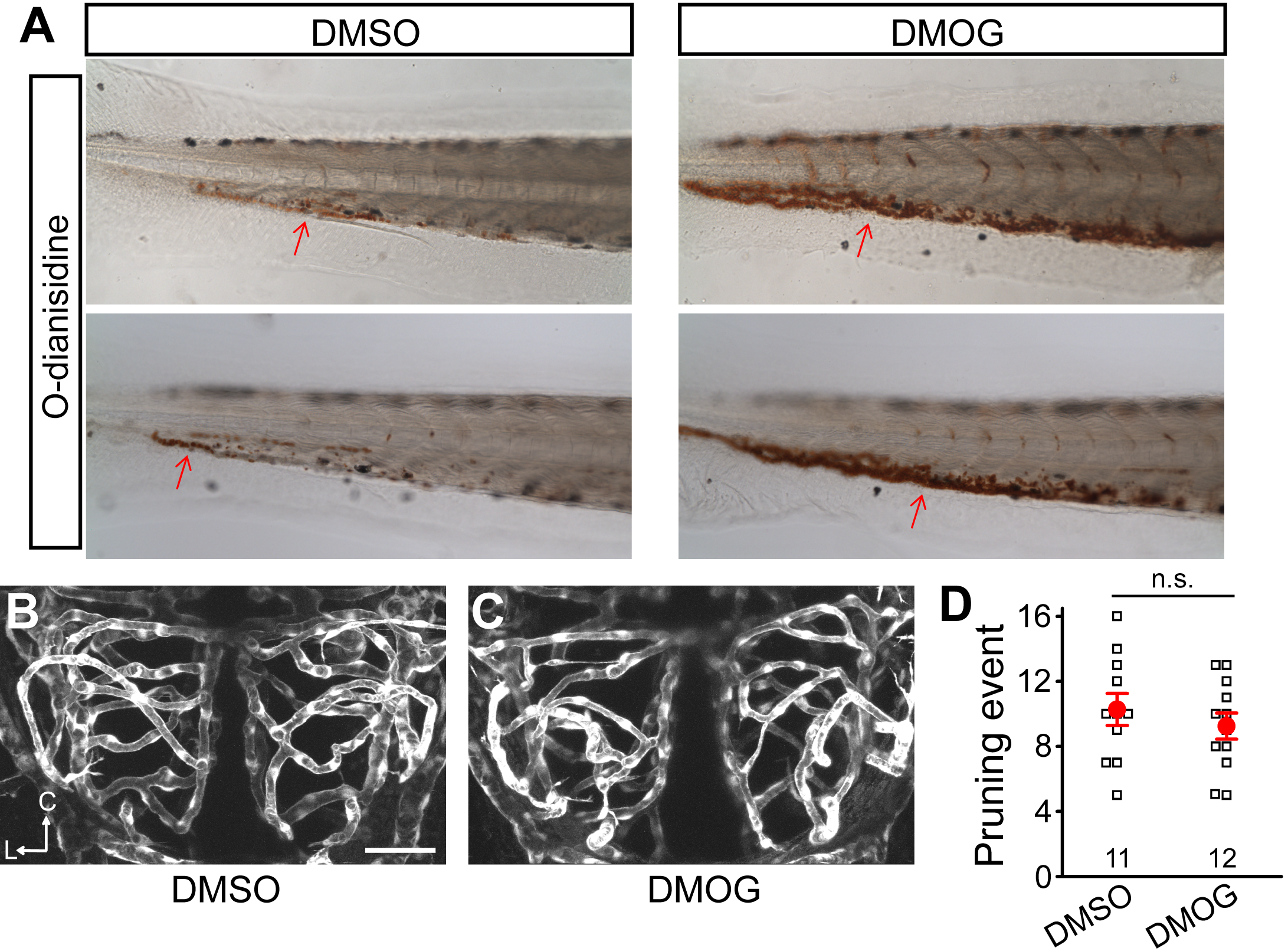

Supplement: Figure S16 — Effects of DMOG treatment on vessel pruning. (A) o-Dianisidine staining showing that DMOG treatment increases the amount of blood cells in treated embryos. DMSO (0.2%) or DMOG (0.2 mM) was bath-applied during 2–3 dpf. (B and C) Effect of DMOG treatment on vessel pruning of larval zebrafish midbrain. Projected confocal images showing midbrain vasculature of DMSO- (B) and DMOG-treated (C) zebrafish larvae at 3 dpf. (D) Average number of vessel pruning events occurring between 2 and 3 dpf in single larval zebrafish midbrain. Each small square in (D) represents the data obtained from single larvae. Scale, 50 µm. n.s., no significance (Student's t test). Error bars, ± SEM. (TIF) [file pbio.1001374.s016.tif]

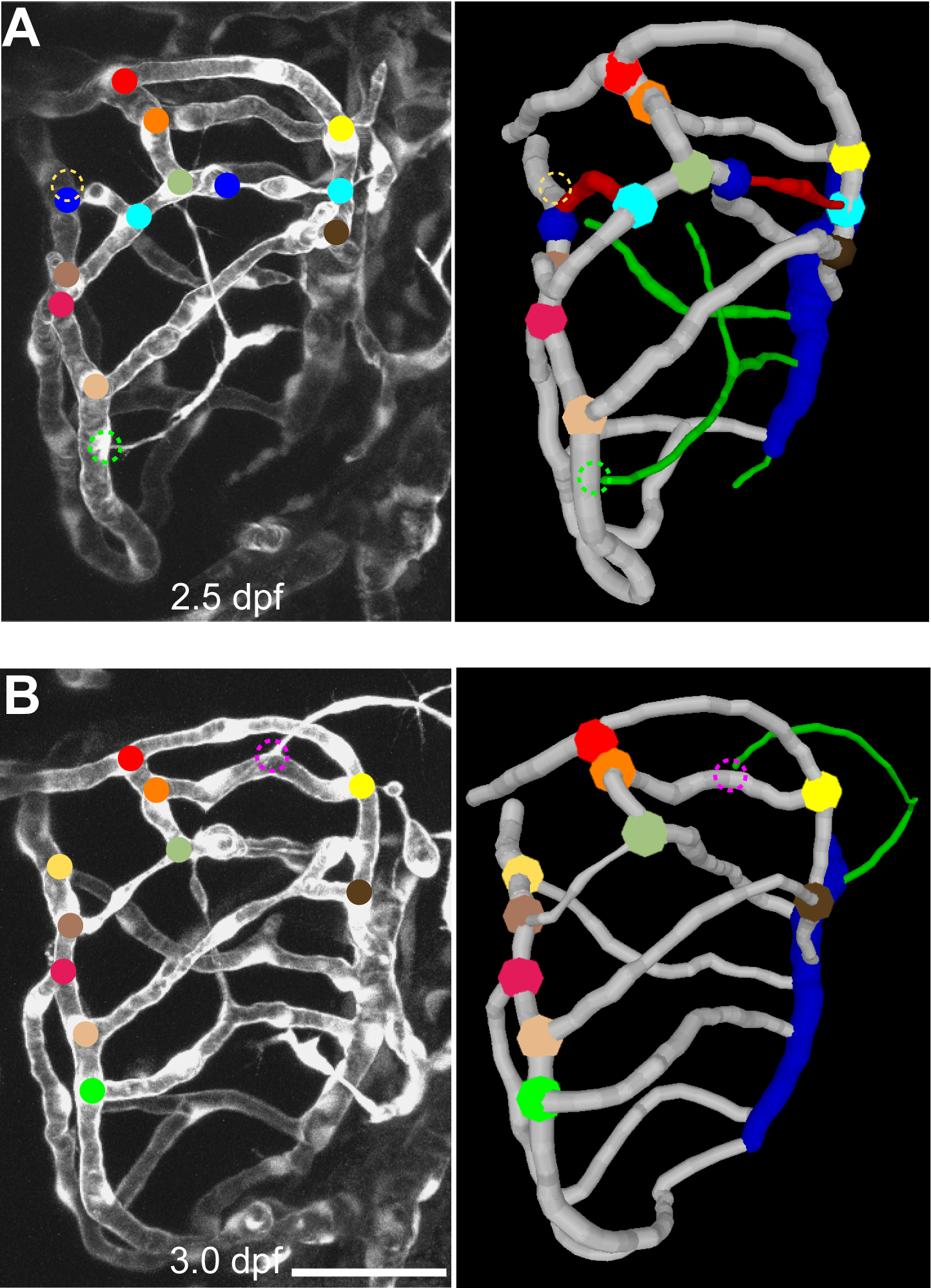

Supplement: Figure S17 — Method for tracing fate of each vessel segment in the midbrain. (A and B) Projected confocal images (left) and 3-D reconstructions (right) of half midbrain vasculature in a Tg(kdrl:eGFP) zebrafish larvae at 2.5 dpf (A) or 3 dpf (B). Colored balls mark different branch points. The dashed circle marks the site at which a branch point will appear at the next imaging time point. The corresponding movies of the 3-D rotation centerlines are shown in Video S7. Green, newly formed segments; red, pruning segments; blue, CVP. Scale, 50 µm. (TIF) [file pbio.1001374.s017.tif]

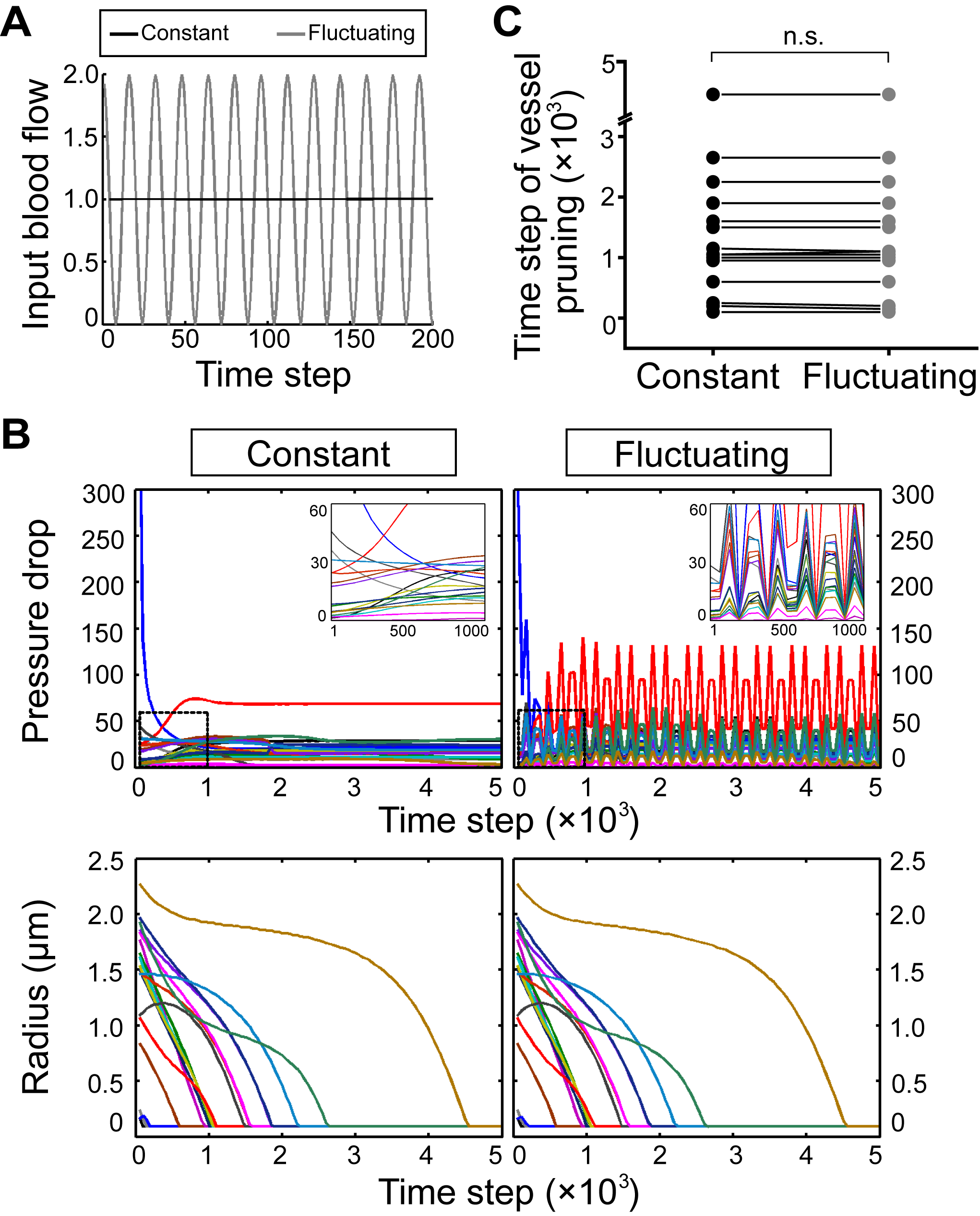

Supplement: Figure S18 — Numerical simulation with constant and fluctuating flow. (A) Diagram of constant (black) and sinewave-like fluctuating blood flow (gray line) with the same mean value. (B) Simulated changes in the pressure drop (top) and radius (bottom) of predicted pruned vessel segments (color lines) when the constant (left) or fluctuating flow (right) is inputted into the mathematical model. The data obtained from each segment are marked with a distinct color. (C) Comparison of time steps required for the completion of vessel pruning. The data obtained from the same vessel segments are connected by a line. n.s., no significance (paired Student's t test). (TIF) [file pbio.1001374.s018.tif]
